# Computational Insights into Materials and Interfaces for Capacitive Energy Storage

**DOI:** 10.1002/advs.201700059

**Published:** 2017-04-24

**Authors:** Cheng Zhan, Cheng Lian, Yu Zhang, Matthew W. Thompson, Yu Xie, Jianzhong Wu, Paul R. C. Kent, Peter T. Cummings, De‐en Jiang, David J. Wesolowski

**Affiliations:** ^1^ Department of Chemistry University of California Riverside CA 92521 United States; ^2^ Department of Chemical and Environmental Engineering University of California Riverside California 92521 United States; ^3^ State Key Laboratory of Chemical Engineering East China University of Science and Technology Shanghai 200237 P. R. China; ^4^ Department of Chemical and Biomolecular Engineering Vanderbilt University Nashville Tennessee 37235 United States; ^5^ Center for Nanophase Materials Sciences Oak Ridge National Laboratory Oak Ridge Tennessee 37831 United States; ^6^ Computer Science and Mathematics Division Oak Ridge National Laboratory Oak Ridge Tennessee 37831 United States; ^7^ Chemcial Sciences Division Oak Ridge National Laboratory Oak Ridge Tennessee 37831 United States

**Keywords:** electric double layers, electrolytes, joint density functional theory, molecular simulations, porous materials, supercapacitors

## Abstract

Supercapacitors such as electric double‐layer capacitors (EDLCs) and pseudocapacitors are becoming increasingly important in the field of electrical energy storage. Theoretical study of energy storage in EDLCs focuses on solving for the electric double‐layer structure in different electrode geometries and electrolyte components, which can be achieved by molecular simulations such as classical molecular dynamics (MD), classical density functional theory (classical DFT), and Monte‐Carlo (MC) methods. In recent years, combining first‐principles and classical simulations to investigate the carbon‐based EDLCs has shed light on the importance of quantum capacitance in graphene‐like 2D systems. More recently, the development of joint density functional theory (JDFT) enables self‐consistent electronic‐structure calculation for an electrode being solvated by an electrolyte. In contrast with the large amount of theoretical and computational effort on EDLCs, theoretical understanding of pseudocapacitance is very limited. In this review, we first introduce popular modeling methods and then focus on several important aspects of EDLCs including nanoconfinement, quantum capacitance, dielectric screening, and novel 2D electrode design; we also briefly touch upon pseudocapactive mechanism in RuO_2_. We summarize and conclude with an outlook for the future of materials simulation and design for capacitive energy storage.

## 1 Introduction

### 1.1 Basics of Capacitive Energy Storage

**Figure 1:**
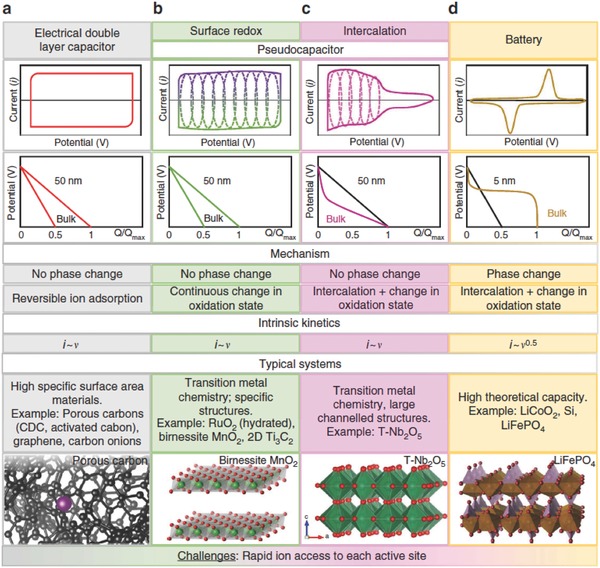
Cyclic voltammetry curves, galvanostatic charging profiles, key mechanism descriptions, and typical systems for: a) electric double‐layer capacitor; b) surface redox pseudocapacitor; c) intercalation pseudocapacitor; d) battery. Reproduced with permission.^3^ Copyright 2016, Nature Publishing Group.

World wide adoption of renewable energy, in the form of solar and wind energy, combined with the electrification of transportation and the proliferation of mobile devices are all driving the need for efficient, cost‐effective electric energy storage devices in sizes ranging from hand‐held to grid‐based. The most commonly used electric energy storage devices are batteries and supercapacitors. A battery stores energy by bulk redox/intercalation reactions, while a supercapacitor stores energy through surface ion‐adsorption or surface redox/intercalation reactions. A battery has high energy density but low power density, while a supercapacitor boasts of high power density due to the fast surface physical and chemical processes.^1^ The global supercapacitor market was $1.2B in 2014 and by some estimates will grow over 20% per year to more than $7B in 2023.^2^ There are two main types of supercapacitors: electric double‐layer capacitors (EDLCs) store electrical energy through formation of electric double layer at the electrode/electrolyte interface (**Figure** **1**a), while pseudocapacitors store electrical energy by reversible surface redox reaction (Figure 1b) or ion intercalation (Figure 1c). EDLCs, pseudocapacitors, and batteries exhibit distinctly different electrochemical behavior in cyclic voltammetry (CV), as shown in Figure 1.^3^

### 1.2 Operating Mechanism of Electric Double‐Layer Capacitors

#### 1.2.1 The Helmholtz Model of an Electric Double Layer

In EDLCs, electrical energy is stored by the formation of an electric double layer (EDL) through the counterion adsorption at the electrode surface. The concept of the EDL originated from the interfacial double layer model in classical surface physical chemistry, first proposed by Hermann von Helmholtz in 1853,^4^ and formed by the coion exclusion and counterion adsorption. According to the Helmholtz model, the differential capacitance of an EDL can be computed by:

(1) $$C\;\;=\;\;\frac{\varepsilon_{r}\varepsilon_{0}A}{d},$$

where *A* is the area of the electrode, ε_*r*_ is the dielectric constant of the electrolyte solvent, ε_0_ is vacuum permittivity, and *d* is the thickness of the Helmholtz layer. Note that this is independent of applied voltage or properties of the electrolyte other than the solvent dielectric constant.

One can use Equation (1) to estimate the areal capacitance (*C_d_* = *C/A*) for a planar electrode. The value of *d* in aqueous electrolytes is several angstroms. Here we assume *d* = 0.3 nm, the size of a typical atom; for an aqueous electrolyte, the value of ε_*r*_ should be about 6 instead of 80 for water in a high electric field.^5^ Thus, *C_d_* is about 18 µF cm^–2^, in good agreement with the average reported values for aqueous electrolyte EDLCs (≈15 µF cm^–2^).^6^ Ionic liquids (IL) usually have a dielectric constant about 10, but their sizes tend to be larger, so we estimate their *C_d_* to be about 10 µF cm^–2^, which is close to the experimental value of ≈11 µF cm^–2^.^6^

#### 1.2.2 The Gouy‐Chapman Model and Beyond

Gouy^7^ in 1910 and Chapman^8^ in 1913 derived, independently, an improved model for differential capacitance, which exhibits dependence on electrode voltage and ionic concentration. The “Gouy‐Chapman” (GC) model introduced the diffuse layer, which describes the ion charge distribution as a function of distance to the electrode surface, and used the Maxwell–Boltzmann statistics to account for the thermal effect. In 1924, Stern proposed combining the Helmholtz model with the Gouy‐Chapman model to describe the electrode/electrolyte interface: a subset of the ions with finite size adsorb on the surface to form the “Stern Layer”, while others form the Gouy‐Chapman diffuse layer. This model is known as the “Gouy‐Chapman‐Stern” (GCS) model.^9^ The GCS model gives a more general description of the electrode/electrolyte interfacial behavior, but is highly simplified. For example, the diffusive ions in the GCS model are treated as point charges. Thus, important ionic features that influence capacitance, such as radii and valences, are ignored. A more rigorous model is necessary, especially for systems utilizing an ionic liquid electrolyte where the diffuse layer and ion steric effect have large influence on the charge capacitive behavior.

**Figure 2:**
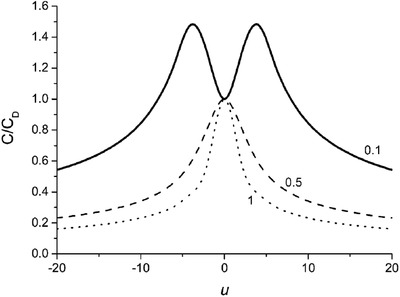
Differential capacitance as a function of electrode potential for different values of the lattice‐saturation parameter γ which is proportional to ion concentration: Bactrian‐camel shape versus bell shape. Reproduced with permission.^10^ Copyright 2007, American Chemical Society.

Fast forward to 2007, Kornyshev proposed a model to describe the interfacial capacitance of the metal/ionic liquid system by solving the “Poisson‐Fermi” equation, which treats the electrolyte by a mean‐field lattice‐gas model.^10^ They derived an analytical expression for the differential capacitance:

(2) $$C\;\;=\;\;C_{0}\cdot \frac{\cosh\left(\frac{u_{0}}{2}\right)}{1+2\gamma sinh^{2}\left(\frac{u_{0}}{2}\right)}\cdot \sqrt{\frac{2\gamma sinh^{2}\left(\frac{u_{0}}{2}\right)}{\ln\left(1+2\gamma sinh^{2}\left(\frac{u_{0}}{2}\right)\right)},}$$

where γ is reduced concentration of ions, *C_0_* is the linear Gouy‐Chapman capacitance defined by *C*_0_ = ε/4*πL_D_*, *L*_D_ is the Debye length, and *u_0_* is surface electric potential in reduced unit. They found that (**Figure** **2**) when the concentration changes from low (γ = 0.1) to high (γ = 1), the differential capacitance curve changes from the “Bactrian‐camel” shape to the “bell” shape, due to the competition between the Stern layer and the diffuse layer, which cannot be captured by the traditional GCS model. This analytical method provides a clear physical picture for the interfacial capacitive behavior. It has been improved recently by adding the short‐range correlation for the ionic liquid electrolyte.^11^ Independently, Bazant et al. derived the same analytical solution (Equation (2)) from solving a modified Poisson‐Nernst‐Planck (MPNP) equation to describe the ion transport during dynamic charging at large bias voltage.^12^, ^13^

### 1.3 An Estimate of How Many Ions Need to be Separated when Charging a Typical EDLC Device

**Figure 3:**
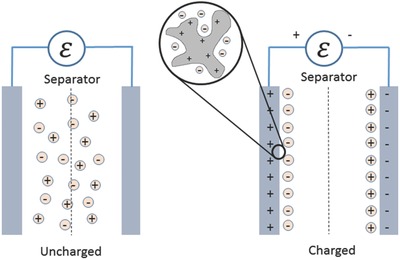
Schematic of an EDLC device consisting of two electrodes in an electrochemical cell.

By pairing two electric double layers at the postive and negative electrodes, one obtains an EDLC. Since the energy is stored through formation of the EDL at the interface, the capacitance per unit weight or specific capacitance of a material generally increases with the specific surface area (SSA). This is why activated carbons with SSA of 1000 to 2000 m^2^ g^–1^ are used in commercial EDLCs. A typical EDLC consists of two symmetric porous carbon electrodes sandwiching a separator, with the electrolyte stored mainly inside the pore volumes of the electrodes (for a commercial device, volume contribution from the separator is negligible). When charged up, the ions separate and form two separate EDLs at the two opposite electrodes (**Figure** **3**). Here we present a simple phenomenological estimate of how many ions are separated when charging an EDLC device to give the readers a general idea of the degree of ion separation when cycling an EDLC device. A typical organic‐electrolyte activated‐carbon‐electrode EDLC has a specific capacitance of about 100 F g^–1^ based on the weight of carbon. Suppose 1 g of carbon for the positive electrode is charged to 1.5 V, while 1g of carbon for the negative electrode is charged to –1.5 V. This leads to a surface charge of +150 C on the positive electrode and –150 C on the negative electrode. The typical pore volume of activated carbon is 0.90 cm^3^ g^–1^ and a typical concentration of an organic electrolyte is 1 m of tetraethylammonium tetrafluoborate (TEABF_4_) in acetonitrile. Hence the total numbers of available ions in this EDLC are 174 C of cations and 174 C of anions. In other words, ion separation is close to 90% when the EDLC is fully charged: the majority of the anions move to the positive electrode, while the majority of the cations move to the negative electrode. The typical thickness of an EDLC electrode is about 200 µm coated on two sides of a current collector (such as Al foil), so the typical length of ions traveled during cycling of an EDLC should be about 100 µm on a timescale of seconds. Thickness of a typical separator is about 40 µm, so we have neglected it in this simple estimate.

### 1.4 Recent Experimental Advances in EDLCs

The most widely used electrode materials for EDLCs are porous carbon‐based materials,^14^, ^15^ such as activated graphene oxide,^16^ activated carbons (AC),^17^ carbide‐derived carbons (CDC),^18^, ^19^, ^20^ carbon nanotubes (CNT),^21^, ^22^, ^23^ onion‐like carbons (OLC)^24^, ^25^ and graphene.^26^, ^27^ The gravimetric capacitance of these carbon materials is sensitive to their structure, especially the porosity and SSA. The pore size can also greatly affect the ion partitioning and packing inside the pores, which may cause a large change in capacitance. The relationship between the pore size and the capacitance of ionic liquids has been investigated experimentally by Simon and Gogotsi.^28^, ^29^, ^30^ This important work reveals the pore size‐dependent capacitance and suggests that the capacitance maximum can be achieved by optimally matching the pore size and ion size. Carbon nanotubes (CNT) have been used as novel electrode materials in EDLCs.^31^, ^32^ The reported capacitance of single‐wall CNT (SWCNT) is 180 F g^–1^ (or ≈14 µF cm^–2^) with an aqueous electrolyte,^22^ and the SSA is estimated to be 1315 m^2^ g^–1^.^33^ Onion‐like carbons have also been reported as promising EDLC electrode materials exhibiting very large power density at discharging rate of up to 200 V s^–1^.^24^, ^34^ Moreover, graphene's unique electronic structure has large influence on the charge capacitive behavior.^17^, ^26^

### 1.5 Experimental Techniques for Characterizing Electric Double Layer

**Figure 4:**
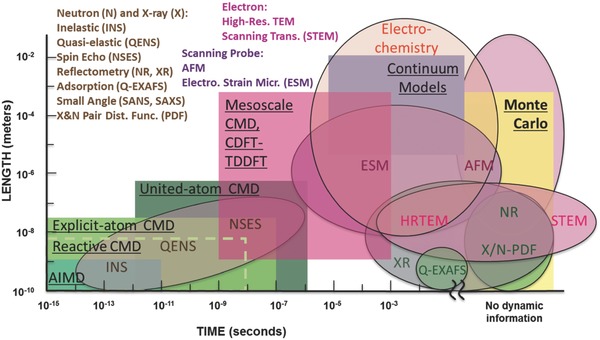
The length and time scales of different simulation and experimental techniques. CMD: classical molecular dynamics; AIMD: ab initio molecular dynamics; CDFT: classical density functional theory; TDDFT, time‐dependent density functional theory. In this figure ESM stands for electrochemical strain microscopy, while throughout the text it stands for effective screening medium.

Characterizing EDL with various experimental techniques is important in both revealing the underlying physics and validating the theoretical predictions and simulations. In a multiyear center effort,^35^ computational/theoretical modeling methods are combined with complementary experimental characterization tools that probe similar spatial and/or temporal scales, as shown in **Figure** **4**, to shed light on the microscopic mechanism of EDL structure and dynamics. Among the experimental methods, Quasi‐Elastic Neutron Scattering (QENS) is an effective technique to study microscopic dynamics on the time scale of pico‐seconds to nano‐seconds for spatially constrained diffusion and long‐range translational diffusion of ions inside porous carbons.^36^, ^37^ Another important tool to probe the solid/liquid interfacial structure on a flat substrate is X‐ray reflectivity, which can provide the structural change of the electrolyte at the interface with sub‐angstrom resolution and under applied potential.^38^, ^39^, ^40^

Electrochemical Strain Microscopy has been developed to explore dynamic electrochemical processes, such as voltage‐controlled ion transport, intercalation and local electrochemical reactivity at the nanometer and meso scales. In particular, for the intercalation process, Electrochemical Strain Microscopy can clearly measure ion dynamics and electrode structure changes, such as expansion and distortion.^41^, ^42^, ^43^, ^44^ Nuclear Magnetic Resonance (NMR)^45^, ^46^ can measure the structure of ion sorption and configuration in supercapacitor electrodes and also provide the picture of ion dynamics in supercapacitor.^47^ Scanning Probe Microscopy (SPM) can directly measure the structure of EDL through the AFM tip that approaches the surface. By measuring the force during the surface scanning, one can obtain the exact structure of EDL, such as the oscillating ion distribution.^41^, ^48^, ^49^, ^50^ Other experimental probes, not shown in Figure 4 but useful for characterizing EDLs, include Sum Frequency Generation (SFG)^51^ and Second Harmonic Generation (SHG),^52^ which can provide the structural information of carbon at interface, such as molecular vibration and bond stretching. Scanning Transmission Electron Microscope (STEM) is also used to visualize the structure at interfaces.

### 1.6 Important Issues in Capacitive Energy Storage

In general, the energy density of a carbon EDLC is below 10 Wh kg^–1^, while a Li‐ion battery's energy density is about 100 Wh kg^–1^. So for capacitors, the most urgent issue is to improve their energy density so that they can better compete with batteries.^1^ To design materials and interfaces for EDLCs with higher energy density requires a deeper understanding of the factors contributing to the total capacitance of an EDLC.

#### 1.6.1 Classical vs. Quantum Capacitance

**Figure 5:**
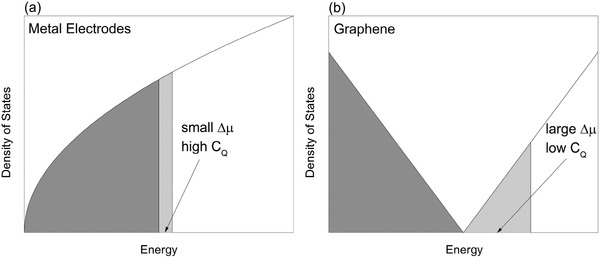
Comparison of charging behavior between a typical metal (a) and graphene (b) from the perspective of the electronic density of states. With the same amount of excess charge, graphene exhibits significantly larger shift in the Fermi level (Δµ) than a typical metal.

From conventional EDL theory, the capacitance of EDLCs stems from the adsorption of counterion at the charged electrode surface. This EDL capacitance can be obtained from classical thermodynamics and electrostatics.^10^, ^53^ Thus, we refer to it as “classical capacitance”, which is not explicitly dependent on the electronic state of electrodes. The macroscopic electrode potential is related to the electron chemical potential μ_*e*_. For an ideal metal electrode, which has extremely large electronic density of states (DOS), adding or removing a few electrons does not cause a significant shift in μ_*e*_ (**Figure** **5**a). Thus, the macroscopic electrode potential change is dominated by the surface potential drop of the EDL. However, for materials with low electronic DOS at the Fermi level, adding or removing the same number of electrons from the electrode can result in a significant potential drop (Figure 5b). This capacitance due to the electrode being a poor metal is called quantum capacitance (*C*_Q_), which is especially important for graphene. Different from C_Q_, the capacitance due to the electrolyte response and EDL formation is defined as EDL capacitance (C_EDL_).

#### 1.6.2 Pore Size, Surface Area, and Pore Geometry

Experimental studies have shown that optimal pore size is related to ion size and can result in a maximum in the capacitance.^28^, ^29^ Theoretical calculations and molecular simulations also verified this phenomenon and predicted that capacitance oscillates with pore size for ionic liquids inside an ideal slit pore.^53^, ^54^, ^55^ However, treating a porous carbon as a single slit pore is problematic because a real porous carbon can exhibit complex features such as a broad distribution and/or a bimodal distribution. These complexities make such systems difficult to model. In particular, ion accessibility and selectivity in a distribution of nanopores can have a significant impact on the macroscopic capacitance.

Specific surface area (SSA) and pore geometry are also important factors in determining EDLC performance. For a flat single layer graphene in contact with electrolyte on both sides, the theoretical SSA is about 2630 m^2^ g^–1^. However, in porous carbon, the SSA is strongly related to the pore geometry. In particular, the curvature is important in porous carbons dominated by micropores.^56^, ^57^, ^58^ Numerical simulation of the Poisson‐Boltzmann (PB) equation has shown that the capacitance of a cylindrical or spherical electrode is radius (*R*_0_)‐dependent when *R*_0_ is small; for large *R*_0_, the capacitance approaches that of a planar electrode.^59^, ^60^ However, an electrode composed of real porous carbon is not in an ideal geometry such as planar, cylindrical or spherical. Thus, the pore size, SSA and pore geometry are coupled together.

#### 1.6.3 Electrolytes

The capacitance can be effectively varied by changing the ion size, valence, and concentration in the electrolyte, which calls for guidance from simulations. For ionic liquids, the cation and anion are usually different in size. As a result, the charging behavior on the two electrodes become asymmetric, as is observed experimentally.^61^ Moreover, solvent is also very important to the energy density of supercapacitor since, for example, it dictates the electrochemical window. Organic solvents offer electrochemical windows up to 3V. Aqueous electrolytes usually have higher capacitance than ionic liquid, but suffer from narrow voltage window of about 1.2V. In addition, the capacitance depends on the scan rate and a promising EDLC is also expected to have large capacitance at fast charging/discharging rate. This requires fast ion transport in response to the electrode charge. The time‐dependent charging behavior can in principle be simulated by molecular dynamics (MD) or solving the Poisson‐Nernst‐Planck (PNP) equation, but difference of many orders of magnitude exists between the simulated time scale (on the order of ns to µs) and the experimental scan rate (seconds).^12^, ^13^, ^62^ A multiscale approach is needed to bridge the gap.

#### 1.6.4 Defects and Functional Groups on Carbon

Introducing surface defects can change the surface structure, atomic bonding and SSA, hence the EDL structure and capacitance.^63^ Simulations need to take into account changes in both the electrode's electronic structure and the EDL structure.^64^ Functionalization is another effective way to modify the graphene electrode and improve the capacitive performance. The mechanism of how functional group influences capacitance can be more complicated than surface defect due to the possible redox reaction caused by functional groups. When the functional group is introduced on a graphene electrode, the electronic structure change can cause *C*_Q_ to change, and the interaction between the functional group and the electrolyte also influences the EDL structure and *C*_EDL_.

#### 1.6.5 EDLCs vs. Pseudocapacitors

The methods to model EDLCs have been well developed. However, currently there is no appropriate method for modeling pseudocapacitance due to the complexity of interfacial redox processes. The pseudocapacitance can be affected by many factors, such as the diffusing behavior of reactant species, EDL at interface, and surface redox kinetics. These issues are difficult to be self‐consistently captured in the same model. So developing a feasible model to capture the pseudocapacitance is urgently needed.

## 2 Classical Methods to Simulate EDLCs

In this section, we briefly review the most commonly used classical methods to simulate EDL and EDLCs. These include classical density functional theory (CDFT), classical molecular dynamics (CMD), and grand canonical Monte Carlo (GCMC). In both CDFT and GCMC, coarse‐grained models are commonly used for the electrolyte, while in CMD all‐atom models are usually employed.

### 2.1 Coarse‐Grained Classical Density Functional Theory

CDFT is a powerful method to simulate the equilibrium properties of complex liquids and soft materials. Both primitive and non‐primitive coarse‐grained models are employed to represent the ionic species, impurities, and solvent molecules in the electrolyte solution.^65^ The model system consists of charged hard spheres for ionic species and a hard‐sphere dimer for solvent molecules. CDFT was used to simulate the EDL structure and capacitance for the electrolyte solution in various pore geometries.^66^, ^67^, ^68^, ^69^, ^70^ The details of the CDFT calculations have been published before.^57^, ^71^, ^72^, ^73^ The density profiles of cations, anions, impurities, and the solvent segments inside the pore can be obtained by minimization of the grand potential, given the number densities of ions and solvent molecules in the bulk, system temperature, pore size, pore geometry, and the surface electrical potential. To illustrate, we may consider an electrolyte system containing spherical cations and anions and solvent molecules represented by two tangentially connected spheres of opposite charge. The grand potential is given by^74^

(3) $$\begin{array}{l} \beta \Omega \left[\rho_{M}(R),\{\rho_{a}(r)\}\right]=\beta F\left[\rho_{M}(R),\{\rho_{a}(r)\}\right]\\ \;\;\;\;\;\;\;\;\;\;\;\;\;\;\;\;\;\;\;\;\;\;\;\;\;\;\;\;\;+\;\int \left[\beta \Psi_{M}(R)-\beta \mu_{M}\right]\rho_{M}(R)dR\\ \;\;\;\;\;\;\;\;\;\;\;\;\;\;\;\;\;\;\;\;\;\;\;\;\;\;\;\;\;+\sum_{a}\int \left[\beta \Psi_{a}(r)-\beta \mu_{a}\right]\rho_{a}(r)dr, \end{array}$$

where β^−1^ = *k_B_ T*, *R*≡*R*(*r*_δ+_, *r*_δ−_) represents two coordinates specifying the positions of two segments in each solvent molecule, μ_a_ is the chemical potential of an ionic species, μ_M_ is the chemical potential of the solvent, Ψ_*a*_(*r*) stands for the external potential for ions, and Ψ_*M*_(*R*) is the summation of the external potential for a solvent molecule. *F* is the total intrinsic Helmholtz energy

(4) $$\begin{array}{l} \beta F\;\;=\;\;\int \left[\ln\rho_{M}(R)-1\right]\rho_{M}(R)dR\\ \;\;\;\;\;\;\;\;\;\;+\beta \;\int V_{b}(R)\rho_{M}(R)dR\\ \;\;\;\;\;\;\;\;\;\;+\sum_{a}\;\int \left[\ln\rho_{a}(r)-1\right]\rho_{a}(r)dr+\beta F^{ex}, \end{array}$$

where *V*_b_ stands for the bonding potential of the solvent molecule and *F^ex^* is the excess contribution due to intermolecular interactions. The detailed expression for each contribution and the numerical details can be found from a previous study.^65^ The mean electrostatic potential can be obtained from the density distributions of the ions by using the Poisson equation. The surface charge density *Q* is obtained from the condition of overall charge neutrality. The differential capacitance *C*_d_ of the EDLs can be calculated as a derivative of the surface charge density *Q* with respect to the surface potential.

Time‐dependent density functional theory (TDDFT) is an extension of the classical DFT to describe dynamic or time‐dependent processes based on the assumption of local thermodynamic equilibrium.^75^, ^76^, ^77^ For ion diffusion in an electrolyte solution near electrodes, TDDFT asserts that the time evolution for the local density profiles of ionic species, ρ_*i*_(*r*,*t*), follows the generalized diffusion equation

(5) $$\frac{\partial \rho_{i}\left(r,t\right)}{\partial t}\;\;=\;\;\nabla \cdot \left\{D_{i}\rho_{i}\left(r,t\right)\nabla \left[\beta \mu_{i}\left(r,t\right)+\beta V_{i}\left(r\right)\right]\right\}\;,$$

where *D_i_* stands for the self‐diffusivity of ion *i*, μ_*i*_(*r*,*t*) is the local chemical potential and can be obtained by a derivative of the intrinsic Helmholtz energy *F* with respect to the density, and *V_i_*(*r*) denotes the external potential from the electrodes. With TDDFT, one can examine the ion dynamics inside the nanopores during charging.

### 2.2 Atomistic Classical Molecular Dynamics

Atomistic molecular dynamics (MD) simulations have been broadly and effectively employed to investigate the structure of EDL and capacitance of EDLCs. The key for MD simulation to accurately capture the fundamental aspects of EDLCs relies on the model used for the electrode/electrolyte interface. To simulate the charged electrodes, two methodologies have been developed: the constant‐charge method and the constant‐potential method. In the constant‐charge method, a partial charge (of equal value) is assigned to each atom on the electrode surface. The potential associated with each value of surface charging is calculated by solving Poisson's equation. Due to the simplicity of implementation, the constant‐charge method has been widely adopted by researchers to simulate electrodes with simplified geometries, such as graphene basal planes,^43^, ^78^, ^79^, ^80^, ^81^ carbon onions,^82^ carbon nanotubes^83^, ^84^ and slit pores.^54^, ^85^ These studies provide insights into the charging mechanism of EDLCs, and the results match well with the experimental results in both capacitance and interfacial structures.^86^

The constant‐charge method neglects possible fluctuations and inhomogeneities in surface charges induced by electrolytes. The constant‐potential method overcomes this drawback and constrains a constant‐potential drop between the two electrodes by redistributing the surface charge over the course of simulation. The charges on the electrode atoms fluctuate in order to satisfy the condition of minimizing the electrostatic energy of the system.^87^, ^88^, ^89^ Researchers have compared these two different methods. Merlet and co‐workers modeled a supercapacitor based on 1‐butyl‐3‐methylimidazolium hexafluorophosphate (BMIM‐PF6) and carbon electrodes, and found that both methods showed quite similar ionic density profiles at low potential drop for the planar surface.^90^ Wang et al. modeled the LiClO_4_‐acetonitrile/graphite EDLCs and drew a similar conclusion.^91^ However, in both studies, the structural difference becomes significant when the potential difference is high (over 5 V).^92^ The constant‐potential method is about one order of magnitude slower than the constant‐charge method,^90^, ^93^, ^94^ but it allows MD simulations to explore more complex and inhomogeneous electrode systems, such as rough surfaces,^95^, ^96^, ^97^ activated carbon^98^ and amorphous carbide‐derived carbons (CDCs),^99^, ^100^, ^101^ where the constant‐charge method is not applicable.

Accurate force fields for the electrolyte are also necessary for MD simulations of EDLCs. Many force fields have been developed for room‐temperature ionic liquids (RTILs), including coarse grained,^102^, ^103^, ^104^ united‐atom,^105^, ^106^, ^107^ all‐atom^108^, ^109^ and polarizable force fields.^110^, ^111^, ^112^, ^113^ Coarse‐grained force fields simplify the whole molecule into a few or even one bead, and are hence useful in reducing computational cost to capture long‐time dynamics^100^ and interfacial properties.^104^, ^105^ But their lack of atomic details for the electrolyte molecule may limit the degree of agreement that can be achieved with experiments.^114^ All‐atom force fields provide a richer representation of the electrostatic interactions and molecule configurations in the EDL. However, the non‐polarizable all‐atom force fields for ionic liquids usually overestimate the ion‐ion interactions, resulting in smaller diffusion coefficients and higher viscosities compared with experimental results. Scaling partial charges has been considered as an effective way to compensate the polarization effect and recover diffusion coefficients and electrical conductivity.^115^, ^116^, ^117^, ^118^, ^119^ Another solution is to use a polarizable force field. The inclusion of electronic polarizability has been shown to not only improve dynamic properties, but also reproduce local ion structures,^111^, ^120^ although at an order of magnitude higher computational cost.^121^

### 2.3 Constant‐Voltage Grand Canonical Monte Carlo

Kiyohara et al. developed a constant voltage GCMC to simulate an EDLC system with planar electrodes and coarse‐grained ions.^122^, ^123^ The Monte Carlo steps include ion insertion, deletion, migration and exchange in the electrolyte as well as charge balance and exchange among electrodes. Given the electrode setup, the bulk electrolyte activity, and the applied potential, many millions of MC steps are performed to reach the equilibrium. Then EDL structure, surface charge, and capacitance can be derived, similar to the process from the CDFT method. The oscillatory behavior in the capacitance of an ionic liquid electrolyte in a slit nanopore shown by CDFT and CMD simulations was successfully reproduced by constant voltage GCMC.^124^, ^125^ Unlike the available CDFT method which is limited to simulate EDLC in one dimension (hence can treat only one pore size in a single simulation), the GCMC method is three‐dimensional and can simulate a multiple‐sized and more realistic porous electrode with both pure and mixture of electrolytes.

## 3 Ab Initio Simulations of the Electrochemical Interfaces

When the electron or the electronic structure plays an important role at the electrochemical interfaces, ab initio simulations are necessary. The electronic effect can originate from the electron‐solvent repulsion at the charged interface and influence the structure and screening potential of the EDL, as revealed by ab initio MD simulations of the aqueous electrolyte/Pt interface.^126^ For carbon‐based EDLCs, the main concern is due to the low electronic density of states at the Fermi level; in this review, we mainly focus on this quantum capacitance (*C*_Q_) effect, which has been briefly introduced in 1.6.1. One can compute the total capacitance (*C*_tot_) by combining *C*_EDL_ from classical simulation and *C*_Q_ from the electronic structure calculation. At the same surface charge density, the total electrode potential drop can be divided into contributions from *C*_Q_ and *C*_EDL_.^80^, ^127^ Methods have also been developed to capture the polarization effect self‐consistently at the electronic‐structure level. Here, the electronic chemical potential shift Δμ_*e*_ is treated as the electrode potential drop and has contributions from band filling/emptying in the electrode and ionic screening in the electrolyte. Currently, there are two methods that can capture the electronic structure of the electrode in contact with the EDL: the effective screening medium (ESM) method and joint density functional theory (JDFT).

### 3.1 Effective Screening Medium (ESM)

**Figure 6:**
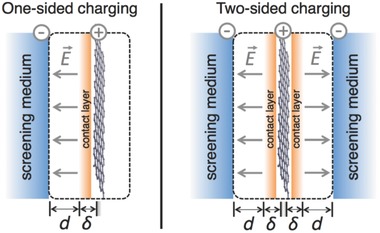
Simulation setup for one‐ and two‐sided charging within the effective‐screening‐medium (ESM) method (shown here for a positive bias). Reproduced with permission.^129^ Copyright 2015, American Physical Society.

The original idea of ESM was proposed by Otani et al. to achieve first‐principles calculation of charged surfaces and interfaces taking into account ionic screening.^128^ In the ESM method an infinite dielectric medium is inserted at the boundary of the periodic cell that can ideally screen all the excessive charge in the periodic cell through setting a mirror charge at the dielectric boundary (**Figure** **6**).^129^ The position and dielectric constant of the ESM are adjustable, which can influence the electrostatic potential of the whole system. The total energy functional includes the electrostatic interaction between the electronic system and the ESM; the electrostatic potential is obtained by solving the Poisson equation. ESM can capture the polarization effect on the electrode from ionic screening of electrolyte. The thickness of the vacuum slab between the screening medium and the electrode is given, so the vacuum part can be regarded as a dielectric capacitor with ε_*r*_ =  1 and *C_vac_* =  *εA*/*d*. Here, *A* is the surface area and *d* is the vacuum slab thickness. By subtracting the contribution of the vacuum slab from Δμ_*e*_, one can obtain the potential drop due to the electrode and then compute *C*_Q_. However, there are still several limitations in ESM: first, the mirror charge in ESM cannot be used to describe the EDL in the electrolyte because the thermal behavior and ion diffusion are not included in the Poisson equation; second, the liquid response to the electrode charge cannot be captured in ESM. Thus, ESM is more applicable to study the electrode capacitive behavior.

### 3.2 Joint Density Functional Theory (JDFT)

#### 3.2.1 Theoretical Framework

The concept of Joint Density Functional Theory (JDFT) was originally proposed by Arias, aimed at solving the solid/liquid interface self‐consistently.^130^ The philosophy of JDFT is treating the solute by electronic DFT and solvent by classical DFT. By minimizing the total free energy, one can obtain the equilibrium state of the solute/solvent system. The free energy functional in JDFT formalism can be written as^131^

(6) $$\begin{array}{l} A_{\mathrm{JDFT}}\;\left[n,\left\{N_{\alpha }\right\},V\left(r\right)\right]=A_{HK}\;\left[n\right]\\ \;\;\;\;\;\;\;\;\;\;\;\;\;\;\;\;\;\;\;\;\;\;\;\;\;\;\;\;\;\;\;\;\;+\;A_{\mathrm{lq}}\left[\left\{N_{\alpha }\right\}\right]\;+\;\Delta A\left[n,\left\{N_{\alpha }\right\},V\left(r\right)\right]. \end{array}$$

*A*_HK_ is the electronic energy functional from Hohenberg‐Kohn Theorems and *A*_lq_ is the classical density functional of the liquid free energy.^131^  Δ*A* is the coupling energy related to the interaction between solute (the electrode) and solvent (the liquid).

#### 3.2.2 Implicit solvation and linear polarizable continuum model

Besides classical DFT, the solvation effect and the electrolyte response in JDFT can be more easily described by implicit solvation with linear response approximation. For example, the total free energy functional of an electronic system solvated in the linear polarizable continuum model (Linear PCM) can be written as:^132^

(7) $$\begin{array}{l} A\left[n\left(r\right),\;\phi \left(r\right)\right]=A_{\mathrm{TXC}}\left[n\left(r\right)\right]\;\\ \;\;\;\;\;\;\;\;\;\;\;\;\;\;\;\;\;\;\;\;\;+\int d^{3}\;r\{\phi \left(r\right)\left[n\left(r\right)-N\left(r,\left\{Z_{I},R_{I}\right\}\right)\right]\\ \;\;\;\;\;\;\;\;\;\;\;\;\;\;\;\;\;\;\;\;\;-\frac{\varepsilon \left(r\right)}{8\pi }|\nabla \phi \left(r\right){|}^{2}-\frac{\varepsilon_{b}\kappa^{2}\left(r\right)}{8\pi }{\left[\phi \left(r\right)\right]}^{2}\}. \end{array}$$

*A*_TXC_ is the single particle Kohn‐Sham kinetic energy plus exchange‐correlation energy. The second term on the right‐hand side is the electrostatic energy of electron and nuclei in electrostatic field. The last two terms correspond to the energy of the electric field in the dielectric medium and the energy contributed by electrolyte ions described by the Debye‐Hückel theory. In this equation, *n*(*r*) is the electron density of the explicit system, *N*(*r*,{*Z*_I_,*R*_I_}) is the nuclei density and φ(*r*) is the total electrostatic potential including Hartree potential and solvation contribution. The local dielectric constant ε(*r*) and inverse Debye length κ(*r*) are determined from the local solvent density, cavity function, bulk dielectric constant (ε_*b*_) and the inverse Debye screening length in the bulk fluid (κ_*b*_).^133^ The equilibrium state of the solute/solvent system is given by minimizing the total free energy functional with respect to *n*(*r*) and φ(*r*).^132^ Advanced solvation models have been developed for JDFT, including non‐local effects,^134^ nonlinear dielectric response,^135^ spherically averaged liquid susceptibility *ansatz* (SaLSA), and charge‐asymmetric nonlocally determined local‐electric (CANDLE) solvation model.^136^, ^137^ SaLSA and CANDLE can capture the nonlocal dielectric response of polar solvent.^138^

#### 3.2.3 Electrochemical Simulation by JDFT

**Figure 7:**
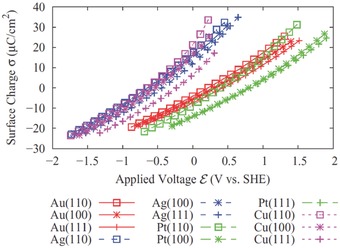
Charge‐potential curve of different metal surfaces from joint DFT (JDFT) calculation with Linear polarizable continuum model (PCM) electrolyte. Reproduced with permission.^132^ Copyright 2012, American Physical Society.

In a two‐electrode electrochemical cell, the electrode potential μ (the voltage) of the working electrode is defined by the energy required to move one electron from the working electrode (electronic chemical potential at μ^*W*^) to the reference electrode (electronic chemical potential at μ^*R*^): so ε = μ^*R*^ − μ^*W*^ per the fundamental charge. If we choose zero potential as a reference, then ε = −μ. When the electrode in the simulation is neutral, the calculated μ is related to the potential of zero charge (PZC). By plotting the calculated PZC and experimental PZC of different metal surfaces, one can extrapolate the potential of standard hydrogen electrode (SHE). **Figure** **7** shows the JDFT‐simulated curves of surface charge density vs. the electrode potential (vs. SHE) for various metal surfaces with Linear PCM.

## 4 EDL Capacitance Inside Nanopores

Having introduced the simulation methods, now we can review how they have been applied to understand capacitive energy storage. The most interesting feature of an EDLC is the structure and capacitance of the double layer confined inside the nanopores. In this section we focus our narrative on the double layer confined in different pore size/geometry from classical simulations, while leaving the electrode chemistry for the next section.

### 4.1 Capacitance vs. Pore Size

#### 4.1.1 Classical DFT Perspective

Despite the fact that a great variety of porous carbons have been utilized in EDLCs, the effects of the pore geometry on the EDL structure is not well understood.^114^ At the heart of the issue is the question: What is the microscopic structure of porous electrodes and how does the capacitance of EDLCs depend on the electrode pore geometry and electrolyte composition? Whereas practical porous electrodes involve micropores with complicated morphology and pore size distributions,^101^, ^139^ theoretical modeling of EDLCs is mostly based on simplistic models to represent the pore geometry and the electrolyte‐electrode interactions.^140^ Specifically, three types of electrode structures are commonly used in theoretical investigations:^141^, ^142^, ^143^ (i) planar surfaces (e.g., a flat surface or slit pores); (ii) cylindrical pores with their concave inner surfaces or cylindrical particles with their convex outer surfaces (e.g., carbon nanotubes); (iii) spherical surfaces (e.g., onion‐like carbons). The slit and cylindrical pore models are conventionally used for porous materials characterization.^144^ Recent simulations and experiments indicate that both the pore size and geometry play an important role in determining the capacitance of EDLCs.^145^, ^146^, ^147^, ^148^ An important question is whether this behavior based on the slit pore or solid particles is generally valid for realistic carbon electrodes.

**Figure 8:**
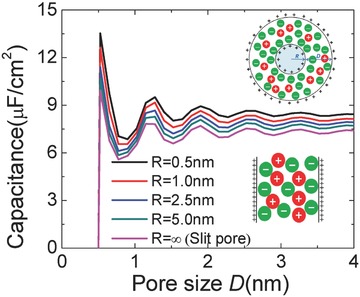
Integral capacitance vs. pore size (D) for a coarse‐grained ionic liquid electrolyte (0.5 nm in diameter for both cation and anion) confined between two spherical shells of different inner radii (R). Reproduced with permission.^57^ Copyright 2016, American Chemical Society.

CDFT is an ideal computational tool for examining the pore size and geometry effects, as it is computationally efficient and applicable over a wide range of pore sizes ranging from that below the ionic dimensionality to mesoscopic scales. To take into account both the pore size and the curvature, a spherical‐shell pore model is used (**Figure** **8** top inset). The EDL capacitance exhibits oscillatory dependence on the pore size for different inner core radii (Figure 8), as observed in previous CDFT and CMD simulations of ionic liquids in slit pores.^53^, ^54^ The distance between neighboring peaks (or valleys) is approximately 1.5 times the ion diameter. The oscillatory variation of the integral capacitance is closely related to the layering structures of ion distributions near the charged surfaces.^53^ As the inner radius *R* decreases, the capacitance increases significantly, because a smaller inner core radius results in more counterions in the pore thus a larger capacitance. This work suggests the significant role of convex surfaces for the synthesis of new porous electrodes to optimize EDLC performance. In particular, the spherical shell model provides a simple yet generic description of both pore size and curvature. The curvature effect on capacitance was also found in CMD simulations of the idealized carbon onion electrodes with the ionic liquid electrolyte.^149^

**Figure 9:**
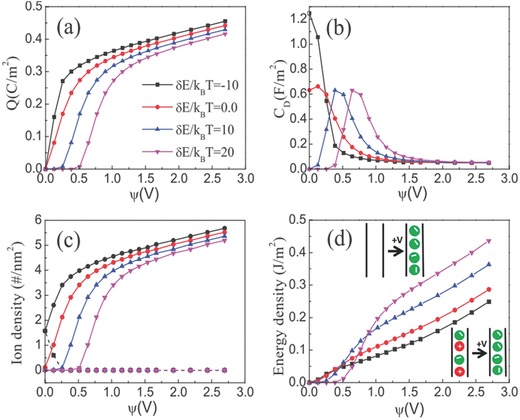
a) Surface charge density, b) differential capacitance, c) average cation and anion densities inside the nanopore, and d) the energy stored per unit of surface area for an EDLC containing a 1.0 m organic electrolyte from coarse‐grained classical DFT. Pore size is *D* = 0.6 nm; both cations and anions are at 0.5 nm in diameter. *δE* represents the ion transfer energy from the bulk reservoir into the nanopore. In (c), the solid lines are average densities inside the pore for the counterions, and the dashed lines are for the coions. Reproduced with permission.^154^ Copyright 2016, IOP Publishing.

Energy storage via electrosorption depends not only on the electrode pores and ionic species but also on specific interactions between mobile ions and the surface of electrode materials. Recently, Kondrat and Kornyshev found that the capacitive performance is sensitive to the ion affinity with nanopores: their theoretical results show electrodes with ionophobic nanopores may have slightly lower, the same, or even higher energy storage capacity than the ionophilic ones, all depending on the electrode voltage.^150^, ^151^, ^152^ It has also been shown that the charging kinetics of an empty ionophobic nanopore is much faster than that of an ionophilic nanopore at similar conditions.^151^, ^153^ Experimentally, the ionophobicity may be controlled by modifying the surface properties of nanoporous materials or by introducing special functional groups to the ionic species. The effects of non‐electrostatic ion‐surface interactions were examined by CDFT.^154^, ^155^ The ionophobicity of nanopores was controlled by *δE*, the resolvation energy or the energy cost to transfer an ion from the bulk to the slit pore (**Figure** **9**a): negative *δE* promotes adsorption of ions within the pore (viz., an ionophilic pore), while a positive *δE* means an ionophobic pore. Figure 9b indicates that the peak capacitance shifts to a higher potential as the ionophobicity increases, because higher potentials are needed to drive ions into the pore with increased ionophobicity (Figure 9c). Figure 9d shows that, at low electrical potential, the energy density for an ionophilic pore is higher than that of an ionophobic pore, while the trend reverses at high potentials. Hence, the energy stored in the EDLC can be promoted by the ionophobicity only when the electrode voltage is larger than a critical value.^154^

#### 4.1.2 Classical MD Perspective

**Figure 10:**
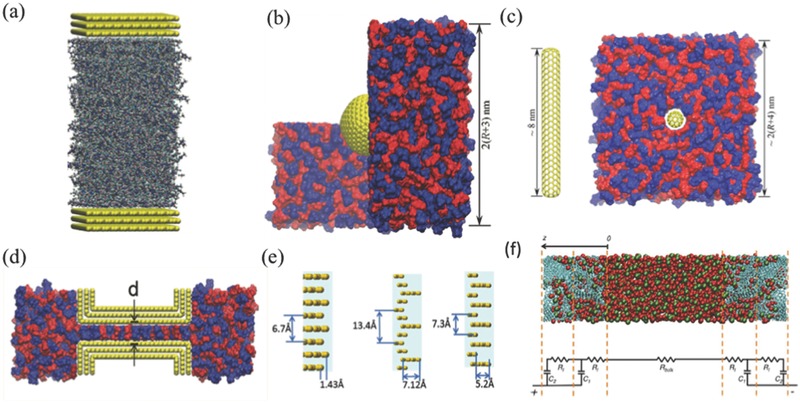
Electrode models used in classical molecular dynamics simulations: a) Planar electrode. Adapted with permission.^78^ Copyright 2014, IOP Publishing. b) Exohedral spherical electrode. Adapted with permission.^149^ Copyright 2012, American Chemical Society. c) Exohedral cylindrical electrode. Adapted with permission.^83^ Copyright 2013, American Chemical Society. d) Slit pore. Adapted with permission.^54^ Copyright 2011, American Chemical Society. e) Rough surface. Adapted with permission.^114^ Copyright 2015, American Chemical Society. f) Realistic pore. Adapted with permission.^100^ Copyright 2014, American Chemical Society.

Although coarse‐grained CDFT is good at capturing most important trends, for atomistic details all‐atom CMD simulations are needed. Many CMD simulations have been devoted to studying the planar electrode (**Figure** **10**a), where differential capacitance is usually found to be bell‐shape or Bactrian‐camel‐shape.^78^, ^107^, ^156^, ^157^ Feng and coworkers built spherical^149^ and cylindrical^83^ electrodes to study influence of curvature effects on capacitance (Figure 10b,c). The differential capacitance was found to be weakly dependent on applied voltage and increase with the electrode curvature.^143^ The slit pore model has been adopted by Feng et al. (Figure 10d) to explain the abnormal increase of capacitance when the pore size is close to ion size for porous carbon electrodes.^54^ Qiao and coworkers also used slit pore models to investigate the charging behavior in subnanometer pores.^62^, ^153^ The simplified electrode models in the CMD simulations usually lead to lower capacitances.^101^ Vatamanu and coworkers showed that the surface roughness (Figure 10e) could increase the energy storage.^95^, ^96^, ^97^, ^158^, ^159^, ^160^ Palmer and coworkers conducted quench molecular dynamics (QMD) simulations to generate porous carbon models by mimicking the thermal quenching of systems of carbon atoms.^139^, ^161^ Their atomistic models for Ti‐CDCs were then used by Merlet et al. to study the capacitance in microporous carbon electrodes (Figure 10f), where quantitative agreement with experimental results was reached.^101^ Moreover, these detailed CDC models allowed the exploration of ion structure and charging mechanism in nanoconfinement.^100^, ^162^, ^163^

### 4.2 Capacitance vs. Electrolyte Composition

Due to their higher voltage window, nonaqueous electrolytes including organic and ionic liquid electrolytes are preferred than aqueous electrolytes for higher‐energy‐density EDLCs. This subsection focuses on the interplay of ions and solvent dipoles in the organic electrolyte and the mixture of ionic liquids when confined in nanopores.

#### 4.2.1 Classical DFT Perspective

**Figure 11:**
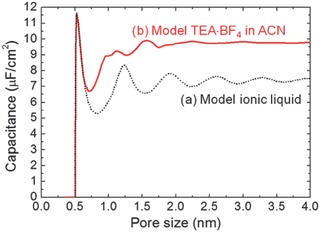
Integral capacitance vs. the pore size for an EDLC with (a) ionic liquid and (b) organic electrolyte (at 1.5 V relative to the bulk electrolyte) from coarse‐grained classical DFT. Reproduced with permission.^71^ Copyright 2012, American Chemical Society.

**Figure 12:**
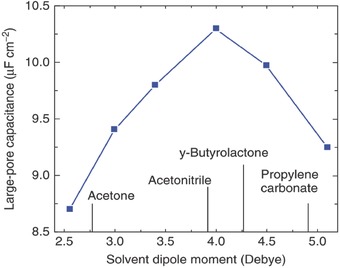
Integral capacitance (of an organic‐electrolyte EDLC with a 4.0‐nm pore) vs. the solvent dipole moment (at 1.5 V relative to the bulk electrolyte) from coarse‐grained classical DFT. Reproduced with permission.^74^ Copyright 2014, Royal Society of Chemistry.

In organic electrolytes, salts such as tetraethylammonium tetrafluoroborate are dissolved in an organic solvent. Acetonitrile (ACN) and propylene carbonate are the most popular solvents. In the coarse‐grained CDFT models, the polar solvent can be represented by a dipole or a connected hard‐sphere dimer, while ions are modeled as charged hard spheres. **Figure** **11** shows how the capacitance changes with the width of the slit pore for both the coarse‐grained organic and ionic liquid electrolytes.^71^, ^72^ In the case of the ionic liquid electrolyte (Figure 11a), the capacitance displays an oscillatory pattern with the slit pore width. The period of the oscillation is about 0.75 nm or 1.5 times the ion diameter. The dampened magnitude converges to about 7.5 µF cm^–2^. The capacitance maximum at the pore size of 0.5 nm or same as the ion diameter agrees with the experiment.^29^ The curve for the organic electrolyte (Figure 11b) also displays such a maximum at the pore size close to the ion diameter but shows a roughly constant capacitance beyond 1.0 nm or twice the ion diameter. Moreover, Figure 11 shows that at the large pore size (≈ 4 nm), the model organic electrolyte has a 30% higher capacitance than the ionic liquid electrolyte. To bridge the gap for the large‐pore capacitance, various dipole moments for the solvent were examined by CDFT:^74^ it was found that the weakly polar solvent has a capacitance closer to that of an ionic liquid, while a capacitance maximum can be achieved at an optimal dipole moment of 4.0 Debye (**Figure** **12**).

**Figure 13:**
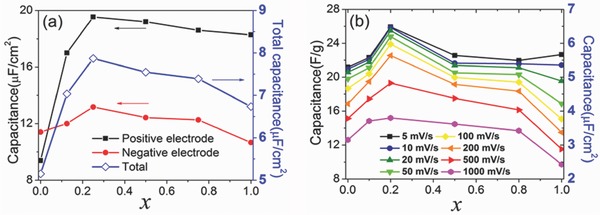
a) Integral capacitance for the positive, negative and total electrodes versus the mole fraction of EMI‐BF_4_ in the EMI‐BF_4_/EMI‐TFSI (*x*) from coarse‐grained classical DFT; operating potential window fixed as 3.0 V between two electrodes. b) Experimental integral capacitance versus *x* for a symmetric EDLC operating at 3.0 V at different scan rates. Note that only the right y‐axis can be directly compared between (a) and (b) panels. Reproduced with permission.^73^ Copyright 2016, American Chemical Society.

An effective strategy to increase the EDLC performance is to use IL mixtures as the electrolyte.^61^ However, little is known about how the EDL structure would be affected by the RTIL mixture composition.^164^, ^165^, ^166^, ^167^ CDFT was used to model a IL mixture based on coarse‐grained models for 1‐ethyl‐3‐methylimidazolium bis(trifluoromethylsulfonyl)imide (EMI‐TFSI) and 1‐ethyl‐3‐methylimidazolium tetrafluoroborate (EMI‐BF_4_) which differ in the size of the anion. **Figure** **13**a shows that the capacitance versus composition of the RTIL mixture has a maximum at 25%.^73^ This is because using a mixture allows more counterions to accumulate on the electrode surface while simultaneously excluding more coions from the electrode surface. The combined result yields a net increase in the counterion density and therefore a larger capacitance. Good agreement has been achieved between the CDFT simulation (Figure 13a) and the experiment (Figure 13b).^73^ Some small amounts of impurity (e.g., water, alkali salts, and organic solvents) in RTILs may affect the electrochemical performance, which was examined by CDFT.^168^, ^169^ It was found that with strong binding of impurity to the ionic species, the RTIL/impurity mixture can lead to an enhanced capacitance oscillation. In certain‐sized pores, a significant increase in the capacitance can be obtained.

#### 4.2.2 Classical MD Perspective

**Figure 14:**
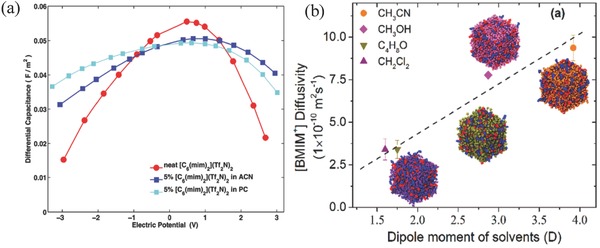
Solvent effect on the ionic liquid electrolyte from all‐atom classical MD simulations: (a) differential capacitance of a dicationic ionic liquid on the graphite basal plane with added solvents (acetonitrile, ACN; propylene carbonate, PC) in 5% molar ratio: [C_6_(mim)_2_](Tf_2_N)_2_ = 1‐hexyl‐3‐dimethylimidazolium bis[(trifluoromethyl)sulfonyl]imide; Reproduced with permission.^171^ Copyright 2014, IOP Publishing. (b) cation diffusivity in 1‐butyl‐3‐methyl‐imidazolium bis[(trifluoromethyl)sulfonyl]imide, [BMIM^+^][Tf_2_N^−^], in solvents of different dipole moments at 50 wt%. Reproduced with permission.^173^ Copyright 2017, American Chemical Society.

Ionic liquids often suffer from high viscosity, and organic solvents can be added to improve the transport properties. From their all‐atom CMD simulations, Feng et al. found strong layering and ordering of solvent molecules near neutral surface and distinct contact adsorption of counterions near the charged surface that cannot be described by the Helmholtz model.^148^ They further constructed a model called “counter‐charge layer in generalized solvents” to describe the structure and capacitance of planar electrode immersed by mixture of RTILs and organic solvents.^170^ Their model predicted that the integral capacitance would increase by less than 10% as the mass fraction of acetonitrile (ACN) in the mixture increases from 0 to 50%. This slight increase of capacitance was confirmed for dicationic ionic liquids in organic solvents.^171^ For differential capacitance, CMD simulations show that the bell shape becomes more diffused (**Figure** **14**a) with the addition of polar solvents. In addition, the solvent helps alleviate the size‐asymmetry effect of ions, generating more symmetric differential‐capacitance curve for positive and negative electrodes.^172^ Vatamanu et al. also reported that the differential capacitance for solvated ILs is weakly dependent on surface roughness.^96^ Recently, Thompson and coworkers combined MD simulations with neutron scattering and reported that the diffusivity and conductivity of an ionic liquid has a positive correlation with the dipole moment and concentration of the organic solvent added, as shown in Figure 14b.^173^ Furthermore, Zhang et al. studied the effect of solvent polarity on the EDL structure and capacitance of graphene‐based EDLCs.^174^

Many RTILs have melting points above 0 °C,^175^ preventing their uses for low‐temperature applications. Mixing ILs has been proven as a successful strategy to expand the temperature range.^176^, ^177^ MD simulations conducted by Li et al. demonstrated that the binary mixture of RTILs outperformed neat RTILs with wider operation temperature, higher conductivity and favorable differential capacitance.^178^ Experiments have also shown that mixing RTILs extends the voltage window for EDLCs by balancing the charge storage between cathode and anode.^61^ While a considerable number of MD simulations have been conducted to investigate the structural and transport properties of bulk RTIL mixtures, further knowledge of the interfacial structure and capacitive behavior of RTILs needs more MD investigation of these aspects.^179^, ^180^, ^181^

### 4.3 Ion Dynamics in Nanopores

#### 4.3.1 Classical Time‐Dependent DFT Simulation

**Figure 15:**
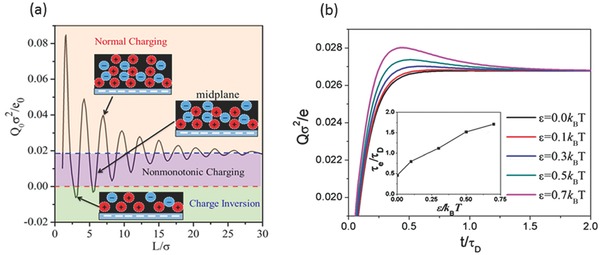
a) Equilibrium surface charge density versus the cell width (L) predicted by classical DFT; Q_0_ is the surface charge density at equilibrium; σ = 0.5 nm is the ion diameter. Reproduced with permission.^187^ Copyright 2014, American Chemical Society. b) Evolution of surface charge density for different strengths of dispersion forces (ε) between ions from TDDFT; inset, time to equilibrium as a function of ε; relaxation time τ_D_ = σ^2^ D^–1^, where D represents ion diffusivity; for a typical ionic liquid, τ_D_ ≈ 5 ns. Reproduced with permission.^188^ Copyright 2016, American Physical Society.

Understanding charging kinetics and ion dynamics inside the nanoporous electrodes is challenging both experimentally and computationally.^100^, ^151^, ^153^, ^182^, ^183^, ^184^, ^185^ Using coarse‐grained models for ions, classical time‐dependent density functional theory (classical TDDFT) is able to describe the charging behavior of ions in a slit pore.^186^ For example, TDDFT predicts that the density profiles of ions can vary with time in a wave‐like fashion for an IL electrolyte,^187^ due to the alternating ion layers near the electrodes (**Figure** **15**a). TDDFT results further show that the dispersion interaction between ions makes the surface charge a non‐monotonic function of time (Figure 15b). However, the dispersion interaction between the electrode and ionic liquid does not change the monotonic evolution of surface charge density.^188^ Both the ion‐ion and the electrode‐ion dispersion interactions increase the duration of the EDL charging process.^188^ TDDFT was also used to examine ion conductance in nanochannels.^189^ In narrow pores with a high gating voltage, the ion conductivity shows an oscillatory dependence on the pore size owing to the strong overlap of electric double layers.^189^

#### 4.3.2 Charging Dynamics from MD Simulation

**Figure 16:**
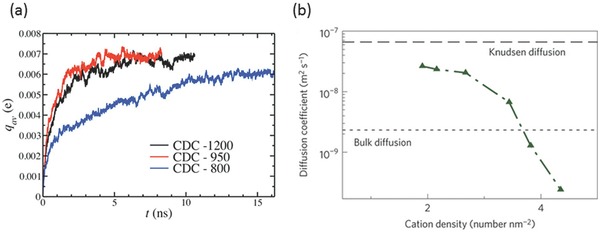
a) Classical MD simulations of the average charge per carbon atom on the electrode vs. time for a coarse‐grained ionic liquid inside the realistic models of carbide‐derived carbons (CDCs) in response to a step electric potential: a sudden potential jump from 0 to 1 V is applied at *t* = 0 between the electrodes; reproduiced with permission.^100^ Copyright 2014, American Chemical Society. b) Classical MD simulations of cation's self‐diffusion coefficient inside a negatively charged ionophobic slit pore at different cation densities; ions are modelled as charged Lennard‐Jones particles of 0.34 nm in diameter; pore size is at 0.53 nm. Reproduced with permission.^153^ Copyright 2014, Nature Publishing Group.

Although nanoporous materials of high SSA are promising electrode materials to enhance energy density, the nanoconfined environment may slow down ion dynamics and thus decrease power density of EDLCs.^190^, ^191^ In their classical MD simulations, Pean et al. used realistic carbon models to study the charging of CDC electrodes.^100^ Based on the response of average charge densities on each electrode atom to a step electric potential (**Figure** **16**a), they found that the ion packing in confinement and the ion‐wall interaction are key factors affecting the ion dynamics in electrified nanoporous electrodes. They further showed that the transmission line model, with inputs such as bulk electrolyte conductivity and capacitance from equilibrium MD simulations, matches well with the non‐equilibrium simulations in the charging/discharging curve.^192^ Diffusion of ions in confinement was found to be slower by one order of magnitude compared to bulk electrolyte and by an additional factor of four for counterions due to their strong electrostatic interaction with the electrode wall.^193^ On the other hand, Kondrat and coworkers used a slit pore model and reported that ion's self‐diffusion is dependent on ion densities and composition and could be higher than the bulk value, as shown in Figure 16b.^153^ Of note, these simulations used coarse‐grained models for the electrolytes.

Using atomistic MD simulations under quasistatic charging conditions, Qiao et al. examined ion dynamics inside charged slit pores.^62^ They found a complex, pore‐size‐dependent charging dynamics: when the pore size is close to one‐layer‐ion thickness, ion diffusivity can exceed the bulk values at certain charge states, while for wider pores, ion diffusivity is below the bulk values. They further conducted cyclic charging/discharging simulations of EDLC based on RTILs and subnanometer slit pore at rates orders of magnitude higher than experimental values.^184^ They found distinct transport properties at pore entrance and pore center: rapid ion migration near the entrance but sloshing dynamics inside the pore. Moreover, the charge storage is dominated by the migration of counterions, deviating significantly from quasistatic charging/discharging conditions. The uneven distribution of ions along the electrode surface was further confirmed for RTIL in cylindrical nanopores.^194^ Electroneutral region, consisted of trapped coion/counterion pairs, was found as a result of rapid overfilling of counterions.

## 5 Quantum Capacitance of Carbon EDLCs

Besides EDL structure and pore size/geometry, the next important factor in EDLCs is the electrode's electronic structure and chemistry. The electronic structure for an EDLC electrode is typically dictated by the electronic density of states (DOS) near the Fermi level. A limited DOS contributes to the charging of an EDLC unfavorably due to the band filling/emptying, leading to quantum capacitance, which is a focus of discussion in this section.

### 5.1 Origin of Quantum Capacitance

In early 1970s, Randin and Yeager observed an unexpectedly low capacitance in stress‐annealed pyrolytic graphite electrode in contact with an aqueous electrolyte, even when the electrolyte concentration was high.^195^, ^196^ The measured differential capacitance showed a “U” shape and the capacitance minimum is about 2 µF cm^–2^ at the PZC. In mid‐1980s, Gerischer et al. proposed an interpretation of the capacitance of graphite electrode in relation to its electronic DOS. The basic idea was to divide the capacitance contribution into two parts: (1) the electrolyte contribution from both Helmholtz layer (C_H_) and diffuse layer (C_diff_); (2) the space charge contribution from the DOS of the graphite electrode (C_SC_):^197^, ^198^

(8) $$\frac{1}{C_{expt}}\;=\;\frac{1}{C_{H}}\;+\;\frac{1}{C_{diff}}\;+\;\frac{1}{C_{sc}}.$$

By measuring the total capacitance (C_expt_) and the electrolyte contribution (C_H_ and C_diff_), C_SC_ and then DOS can be obtained. The DOS indirectly measured by this way showed good agreement with the theoretical DOS of graphite. In other words, C_SC_ proposed by Gerischer et al. is closely related to what we now call the quantum capacitance.

The term “quantum capacitance” was first used by Luryi in 1988, as a general physical quantity to describe the DOS‐related charging behavior in 2D materials used for solid‐state electronic devices.^199^ In solid‐state physics, the electrons in metal can be treated as electron gas. Thus, when the metal layer is atom‐thin, it could behave as a 2D electron gas (2DEG) in a quantum well; when charging such a 2DEG system, quantum capacitance (C_Q_) arises due to the limited electronic DOS at the Fermi level. C_Q_ of 2DEG^200^ and graphene have been obtained analytically and used in study of solid‐state devices.^201^ Below we focus our discussion of C_Q_ in the context of EDLCs, i.e., a 2D material in contact with a liquid electrolyte.

### 5.2 Experimental Measurement of Quantum Capacitance

**Figure 17:**
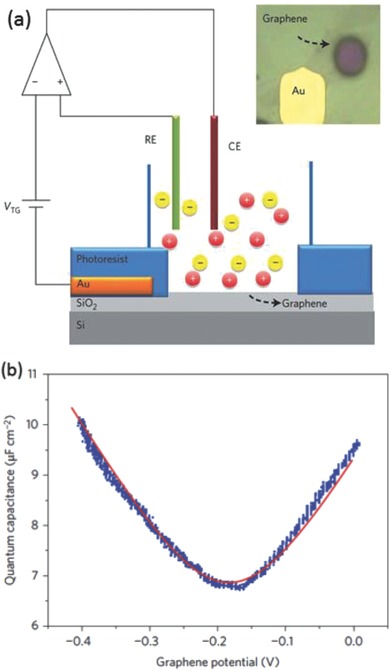
(a) Setup and (b) result of quantum capacitance measurement of graphene against an ionic liquid electrolyte, 1‐butyl‐3‐methylimidazolium hexafluorophosphate (BMIMPF_6_). Reproduced with permission.^202^ Copyright 2009, Nature Publishing Group.

Tao et al. first measured C_Q_ of a single layer graphene in an electrochemical cell that showed good agreement with theoretical prediction (**Figure** **17**).^202^ The measurement is based on the “two‐contribution” model of interfacial capacitance: 1/C_tot_ = 1/C_EDL_ +1/C_Q_. C_tot_ is experimentally measured in the electrochemical cell. C_EDL_ can be roughly treated as a constant from one electrode to another due to the dominant contribution of the Helmholtz layer in aqueous electrolyte. C_EDL_ can be measured by using a metal electrode such as Pt instead of a graphene electrode in the same experimental condition. By subtracting the measured C_EDL_ from C_tot_, one can obtain C_Q_. Using a similar approach, Ruoff et al. studied the layer effect on the capacitance for few‐layer graphene electrodes,^203^ and Downard et al. measured C_Q_ of aryldiazonium modified few‐layer graphene electrodes.^204^

### 5.3 Simulating Quantum Capacitance in Single‐Layer Graphene EDLC

Since C_Q_ arises from the electrode DOS, one can directly compute the electrode's electronic structure and then derive C_Q_. If one assumes that the electronic structure is unchanged during charging, this is called the fixed‐band approximation.

#### 5.3.1 Fixed Band Approximation

Fixed‐band approximation (FBA) is a simple way to simulate C_Q_ in EDLC. Assuming that the Fermi level is rigidly shifted by bias potential φ and DOS is not affected by charging, the excess charge density Q on the electrode can be written in terms of DOS [D(E)] and the Fermi‐Dirac distribution [*f*(*E*)]:

(9) $$Q\;\;=\;\;e\int_{-\infty }^{+\infty }D(E)(f(E)-f(E+e\varphi ))\;dE,$$

so that

(10) $$C_{Q}\;\;=\;\;\frac{dQ}{d\varphi }\;\;=\;\;\frac{e^{2}}{4kT}\;\overset{+\infty }{\underset{-\infty }{\;\int }}D\left(E\right)sech^{2}\left[\frac{E+\varphi }{2kT}\right]dE.$$

For any electrode system, one first computes its electronic DOS by using an electronic‐structure method such as Kohn‐Sham DFT (KS‐DFT) and then uses Equation (10) to obtain C_Q_.^80^ In addition, it is shown that the FBA‐obtained C_Q_ is not affected by the explicit ionic liquid electrolyte.^205^

#### 5.3.2 Relaxed‐Band Approach

**Figure 18:**
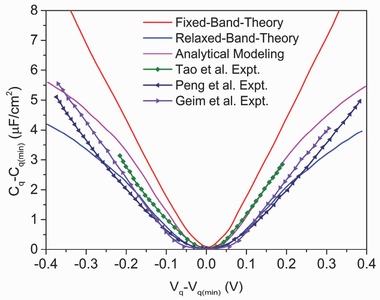
Experimental and theoretical quantum capacitances of single layer graphene. Figure legends from top to bottom are from: Ref. 80, Ref. 129, Ref. 202, Ref. 206, and Ref. 207.

In the relaxed‐band approach, the electronic structure is fully optimized or relaxed after the electrode is charged, hence the name “relaxed band” (RB). One then directly computes the contribution of band emptying/filling, as reflected in the Fermi level shift, to the total potential drop across the interface to compute C_Q_.^129^ Wood et al. simulated C_Q_ of graphene in the RB approach through the ESM method. Their simulated C_Q_ compares better to the experimental measurement (**Figure** **18**), while the FBA curve overestimates. But overall, both approaches can well capture the main trend of graphene C_Q_.

### 5.4 Modeling Total Capacitance of Graphene Electrodes

As discussed above, quantum capacitance is indirectly obtained experimentally from subtraction from the measured total capacitance, while it can be directly computed theoretically. To compare with the experimental total capacitance, the C_EDL_ contribution from classical simulations needs to be combined with computed C_Q_ to predict the total capacitance.

#### 5.4.1 Two Contribution Model

Hwang and coworkers modeled the capacitance of graphene by combining separately simulated C_Q_ from KS‐DFT and C_EDL_ from CMD,^80^ based on two serially connected capacitors. The PZC from CMD simulation is treated as the electrode potential reference and used to set the Fermi level in the KS‐DFT calculation. By summing up the potential drops from C_Q_ and C_EDL_ at the same charge density, one can obtain the total potential drop and total differential capacitance curve. They found that the voltage‐dependent C_tot_ of graphene shows a “U” shape near the PZC and becomes flat when the bias voltage is large. This unique “U” shape of differential capacitance of graphene was also reported by Sun et al. through JDFT simulation that treated the electrode and electrolyte self‐consistently.^208^ In addition, Ruoff et al. reported a similar trend with both experimental evidence and theoretical interpretation.^209^ The “U” shape mainly stems from C_Q_: as shown in Figure 18, C_Q_ of graphene has a “V” shape and a very low value near the Fermi level (PZC), thereby dominating C_tot_ near the PZC.

#### 5.4.2 Doping of Graphene

**Figure 19:**
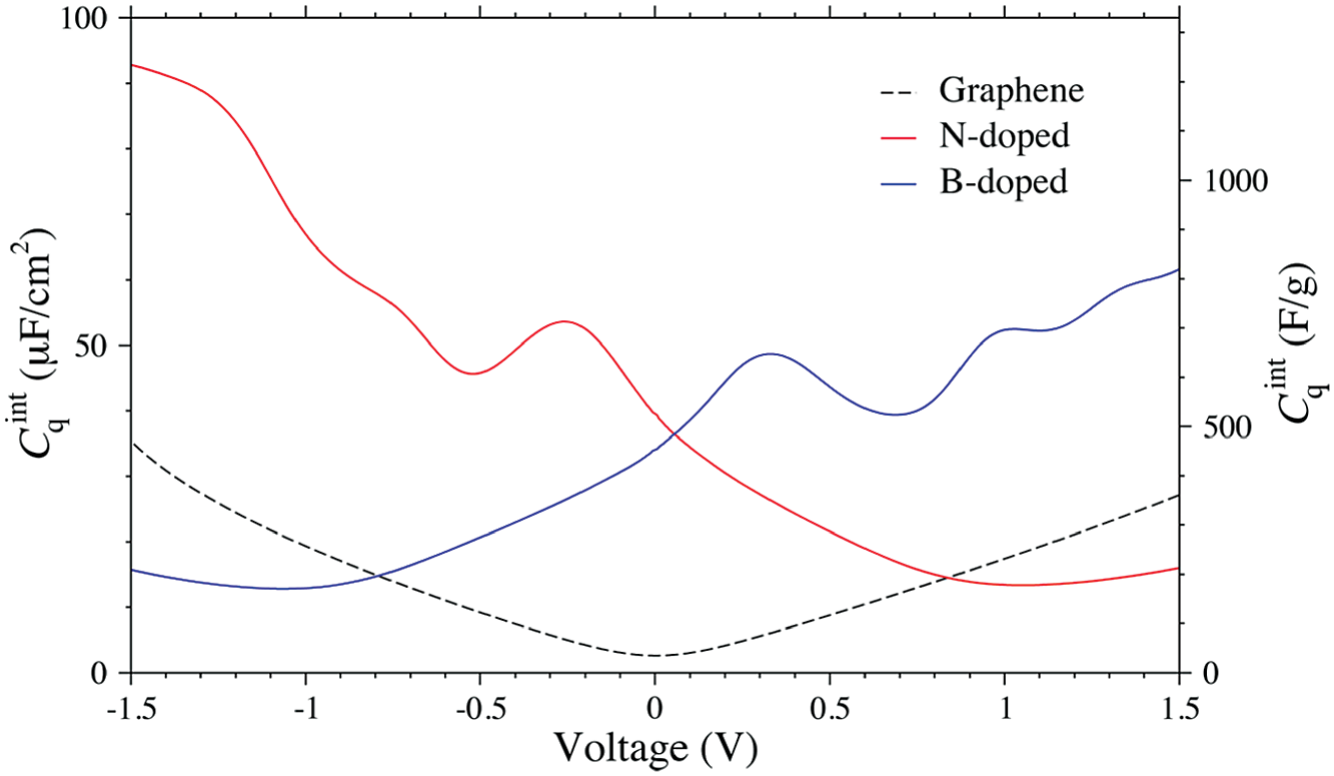
Quantum capacitance of N‐doped and B‐doped graphene. Reproduced with permission.^211^ Copyright 2014, American Chemical Society.

Based on the two‐contribution model, a straightforward approach to increase C_tot_ of graphene materials is to increase C_Q_ by raising DOS at the Fermi level. Hwang et al. showed that N‐doping can greatly increase C_Q_ of graphene and lead to higher C_tot_, which can explain the experimentally observed capacitance increase in N‐doped graphene electrode.^210^ The doping effect on DOS and C_Q_ can be well understood in some simple cases, such as graphitic N and B doped graphene.^211^ Replacing one carbon atom by nitrogen in graphene adds one electron to the conduction band and causes the Fermi level shift to the higher energy, while replacing one carbon atom by boron in graphene removes one electron from the valence band and causes the Fermi level shift to the lower energy. In both cases, the DOS at the new “Fermi level” is larger than that of Dirac Point in graphene, leading to a higher C_Q_ (**Figure** **19**).

**Figure 20:**
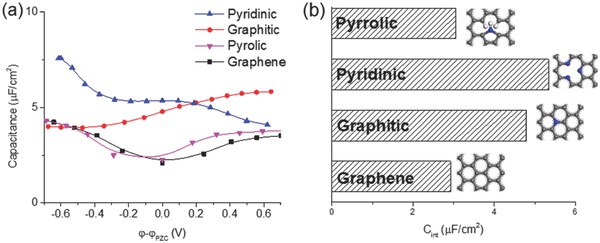
Simulated total capacitance of N‐doped graphene: a) differential capacitance; b) integral capacitance from –0.6 to 0.6 V. Reproduced with permission.^212^ Copyright 2016, Royal Society of Chemistry.

More types of N‐doping were explored later,^212^ including graphitic, pyridinic and pyrrolic. KS‐DFT calculations showed that graphitic and pyridinic nitrogen can greatly increase C_Q_ monotonically with doping concentration, while pyrrolic nitrogen gives a V‐shaped C_Q_, similar to pristine graphene. CMD simulation showed that C_EDL_ is significantly affected by nitrogen doping. C_tot_ from combining the KS‐DFT‐obtained C_Q_ and CMD‐obtained C_EDL_ showed that graphitic and pyridinic N‐doped graphenes exhibit higher C_tot_ than pristine graphene, while the pyrrolic N‐doped graphene has the same C_tot_ as pristine graphene (**Figure** **20**).^212^ Moreover, computed formation energy shows that the relative stability follows pyrrolic > pyridinic > graphitic.^213^ Thus, to effectively improve the capacitance, one should enrich the graphitic and pyridinic nitrogens, and avoid pyrrolic nitrogen. This idea is supported by experimental evidence: Ruoff et al. obtained a U‐shaped differential capacitance on N‐doped carbon and XPS showed that the pyrrolic nitrogen is dominant.^214^ Choi et al. studied the capacitance of N‐doped graphene synthesized by plasma and found that the capacitive performance is dependent on the relative proportion of nitrogen configurations. As the pyrrolic nitrogen content increases, the capacitance decreases, consistent with our theoretical finding.^215^ In addition, N‐doping could yield stronger bilayer formation than pristine graphene,^216^ thereby leading to a potentailly different behavior of capacitance dependence on the layer thickness.

Other dopants have also been explored. Hwang et al. studied C_tot_ of metal‐doped graphene electrode by combining DFT‐obtained C_Q_ and CMD‐obtained C_EDL_.^217^ Two types of configuration were studied: monovacancy and divacancy site. The KS‐DFT study shows that most transition‐metal dopants can greatly increase C_Q_ of graphene and have an asymmetric charging behavior. CMD simulation shows that doping causes only a small increase in C_EDL_. Together, they lead to higher C_tot_. Mousavi‐Khoshdel et al. studied C_Q_ of co‐doped graphene (by N, S, Si, and P) and found that most of them exhibit very large C_Q_, comparing with pristine graphene.^218^

#### 5.4.3 Defect, Curvature and Geometry

**Figure 21:**
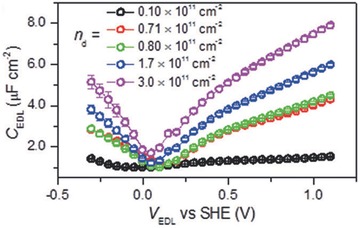
Experimental differential capacitance of a graphene electrode with different topological defect concentrations. Reproduced with permission.^63^

Besides doping effect, the structural modification on carbon itself is also an effective way to improve C_Q_. Wood et al. showed that defect or vacancy can greatly increase C_Q_ of graphene.^211^ Hwang el al. found that topological defects with special geometry shows larger C_Q_ than pristine graphene.^64^ In experiment, vacancies and defects can be created in porous carbons by KOH activation and are expected to have higher capacitance than perfect graphene. Another way of structural modification is surface curvature. Wood et al. showed that strain‐induced ripples can increase C_Q_.^211^ Hwang et al. found that C_Q_ of CNT increases as its diameter decreases.^84^ A strong experimental evidence of defect effect was recently reported, as shown in **Figure** **21**.^63^ The TEM image clearly shows that the synthesized graphene contains some topological defects that can be controlled experimentally. Differential capacitance measurement shows that the capacitance increases with the defect concentration. Geometry effect such as surface wrinkles was also investigated from MD simulation.^219^

### 5.5 Dielectric Screening and Three‐Contribution Model

As discussed in the preceding section, understanding of graphene EDLCs is based on the two‐contribution model that combines C_Q_ from electronic DOS and C_EDL_ from ionic response. This model shows good agreement with the experiment for a single‐layer graphene electrode. However, such a model cannot give a good interpretation on the layer effect for few‐layer graphene (FLG) electrodes. Ruoff et al. measured the capacitance of FLG with different layer number and obtained the Helmholtz layer capacitance (C_H_) of FLG by subtracting calculated C_Q_ from measured C_tot_.^203^ The obtained C_H_ showed strong dependence on the layer number, which cannot be explained by the traditional EDL theory. As it turned out, dielectric screening becomes important in FLG electrodes.

Uesugi et al. studied the capacitance of FLG in IL electrolyte.^220^ They treated the dielectric behavior of FLG classically by using the macroscopic dielectric constant of graphite. Simulated capacitance including the dielectric screening contribution shows good agreement with experimentally obtained layer‐dependent total capacitance.^220^ To take into account the electronic structure of FLG,^221^ Wood et al. applied KS‐DFT with ESM to study the capacitance of graphene and obtained a contribution that lumped quantum capacitance and dielectric screening contribution together.^129^

Although the physical origins of quantum capacitance and dielectric screening are closely related, they can be treated and quantified separately in addition to the EDL contribution by separating their contributions from the total potential drop (φ), leading to a three‐contribution model:^222^

(11) $$C_{tot}\;\;=\;\;\frac{Q}{\varphi_{Q}+\varphi_{Dielec}+\varphi_{EDL}},$$

so that

(12) $$\frac{1}{C_{tot}}\;\;=\;\;\frac{1}{C_{Q}}\;+\frac{1}{C_{Dielec}}+\frac{1}{C_{EDL}}.$$

**Figure 22:**
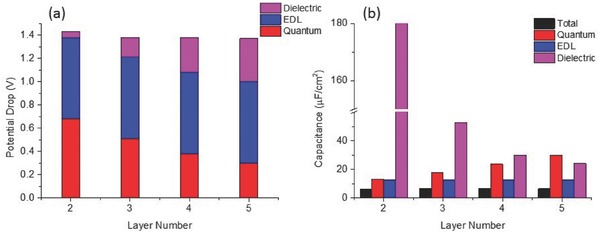
Joint DFT simulations of few‐layer‐graphene electrodes with an implicit solvation model: a) Breakdown of the potential drop into quantum, EDL and dielectric screening contributions with the layer number under the same surface charge density of 9 µC cm^–2^. b) The corresponding integral capacitance contributions. Reproduced with permission.^222^ Copyright 2016, American Chemical Society.

JDFT was used to calculate the capacitance of FLG electrodes in aqueous electrolyte. By separating the electronic chemical potential shift into quantum (C_Q_), EDL (C_EDL_) and dielectric (C_Dielec_) contributions (**Figure** **22**a), one can elucidate how the different parts dominate the total capacitance. C_Q_ from JDFT showed a linear increase with the layer number. The EDL potential drops of FLG are very close to the surface potential drop of a charged Pt surface. The dielectric contribution shows strong dependence on the layer number (*n*) as shown in Figure 22b: C_Dielec_ is very large when n is small and decreases significantly when n increases. Thus, the total capacitance of FLG is dominated by C_Q_ when *n* is low, and limited by C_Dielec_ when *n* >3, while C_EDL_ is roughly constant with *n*.^222^

We note that the above analysis regarding the dielectric contribution focuses on the FLG electrodes for which the thickness is well defined. Single‐layer graphene can also suffer from dielectric screening but its contribution is more difficult to separate from the other contributions due to the difficulty in defining the thickness and the electrode/electrolyte boundary for the single‐layer graphene.

## 6 From Graphene to Amorphous Carbon

Activated carbon electrodes have diverse structure and form, but most theoretical studies still focus on graphene‐based model. Although the slit pore surface of a porous carbon can be simulated by a graphene basal plane, pore mouth is better represented by an edge plane. Moving beyond the graphene‐based models requires simulating the amorphous structure of a carbon electrode. In this section, we will first introduce several recent experimental and theoretical studies on the edge effect in carbon electrode, and then focus on the reverse Monte‐Carlo and MD techniques for modeling structure and EDL capacitance of amorphous carbon electrodes.

### 6.1 Edge Planes vs. Basal Planes

The electrochemical behavior of carbon edge planes has been reported in some experimental studies: Endo et al. investigated the capacitive performance of edge‐enriched porous carbon and found that it has much higher capacitance than traditional porous carbon electrodes, while Bashir et al. indicated that the edge plane of graphene has higher electrochemical activity than basal plane through direct electrochemical measurement on the edge of single graphene layer nanopore.^223^, ^224^, ^225^ A recent work reported by Cen et al. provided a clearer view on the edge effect: as the ratio of graphene edge plane decreases, the measured capacitance shows a significant decrease.^226^ Thus, all of the experimental evidence above shows that introducing more edge sites in carbon EDLCs can improve the capacitance. However, the exact mechanism of edge effect on the capacitance is not clear.

Graphene has two common types of edges: armchair and zigzag. The armchair edge is semi‐conductive, while the zigzag edge is metallic with edge states. Based on the two‐contribution model, Hwang et al. studied the capacitance of graphene edges by combining C_Q_ from DFT and C_EDL_ from CMD and found that C_tot_ increases for edge plane.^227^ However, since the graphene edges also exhibit typical dielectric behavior, it is necessary to include the dielectric screening contribution in total capacitance with the three‐contribution model.^221^ To this end, JDFT was used to study the capacitance of graphene edge planes in contact with an implicit aqueous electrolyte: the electronic chemical potential shift and the electrostatic potential drop across the interface show that armchair and zigzag edges have distinct dielectric screening; the zigzag edge has much higher total capacitance than armchair edge due to its very high C_Q_; CMD simulation was also used to study the influence of surface morphology on the C_EDL_.^228^

### 6.2 Modeling the 3D Structure of Porous Carbon

To simulate activated carbons used in EDLCs, two most commonly used techniques are reverse Monte Carlo (RMC) and quenched molecular dynamics (QMD). Monte Carlo methods of various sophistication have been employed to generate atomistic structures that reproduce either experimental and/or physically realistic radial distribution functions and pore size distributions.^229^, ^230^, ^231^ But Monte Carlo simulation code tends to suffer from poor parallelization that limits the ability to generate large models.^232^, ^233^, ^234^ Another method for generating such structures is quenched molecular dynamics (QMD) simulations. A liquid‐like configuration of disordered atoms at high temperature is used as the initial state. Then, the system is cooled to a lower temperature until a stable solid structure is formed. Because QMD is a molecular dynamics routine, the temperature is tightly controlled with a thermostat and intra and intermolecular interactions are governed by the selected force field. In contrast to RMC simulations, QMD simulations are able to take advantage of recent advances in parallelization and computational efficiency.^235^

**Figure 23:**
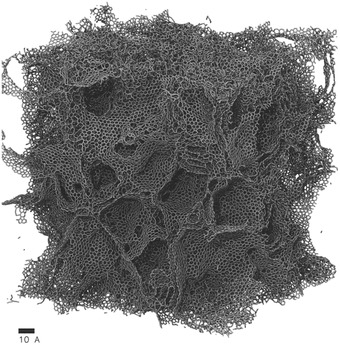
Model of a carbide‐derived carbon (CDC) generated via quenched molecular dynamics with a ReaxFF force field.

Previous QMD studies employed force fields specific to the application, such as amorphous carbon, glass, and metalloids.^139^, ^236^, ^237^, ^238^, ^239^ Using a more rigorous and generalizable force field would improve the validity of the model and allow extensions to various chemical heterogeneities. To this end, the ReaxFF reactive force field was used with a QMD routine to generate atomistic, scalable models for carbide‐derived carbons (CDC). ReaxFF allows bonds to break and form in the bond order formalism.^240^ Unlike other reactive force fields, ReaxFF includes electrostatics and two‐, three‐, and four‐body interactions. Interaction parameters are tuned to thermodynamic data from first‐principles calculations.^240^ A representative structure of this QMD model is shown in **Figure** **23**. A cubic box of length 25 nm includes 200,000 carbon atoms. Nanopores of various sizes are observed. Nanoporous domains are comprised of graphene‐like sheets, which themselves exhibit a mixture of sheet‐sheet stacking and contain defects such as non‐hexagonal rings and curvature.

### 6.3 Charging Inside a 3D Porous Carbon from Constant‐Voltage MD

**Figure 24:**
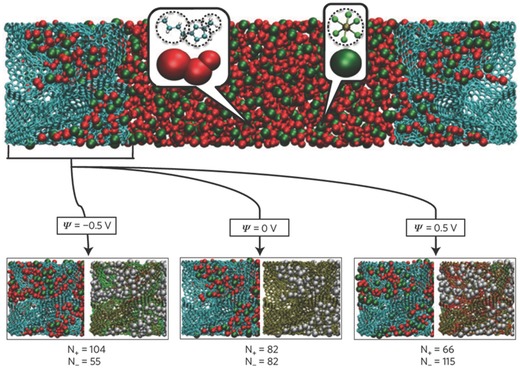
Simulation of an EDLC cell comprising two realistic CDC models at the end with a bulk coarse‐grained ionic‐liquid electrolyte in the middle from a constant‐potential MD simulation. Reproduced with permission.^90^ Copyright 2013, American Chemical Society.

With an appropriate model of porous carbon electrode, one can apply constant‐potential simulation to directly simulate a realistic electrochemical behavior of an electrolyte such as an ionic liquid in the nanopore. Salanne et al. utilized constant‐potential MD to simulate the coarse‐grained IL electrolyte in the nanoporous carbon electrode generated from atomistic QMD simulation of a CDC.^139^ The simulation results provide a clear view on the capacitance increase by the pore size effect, due to the weakening of overscreening (**Figure** **24**).^90^ Similar work also has been reported by Bhatia et al. through constant‐potential MC in a realistic CDC model.^125^

## 7 Modeling Pseudocapacitors

Understanding pseudocapacitors is more challenging due to the complexity of interfacial physics and chemistry. There are two main ways to simulate their behavior: (a) numerical solution and scaling analysis of classical equations (such as PNP and PB equations) to describe the solid/liquid interface with specified boundary conditions; (b) atomistic modeling based on quantum mechanics and molecular mechanics.

### 7.1 Numerical Simulation of Intercalation Pseudocapacitors based on a Continuum Model

**Figure 25:**
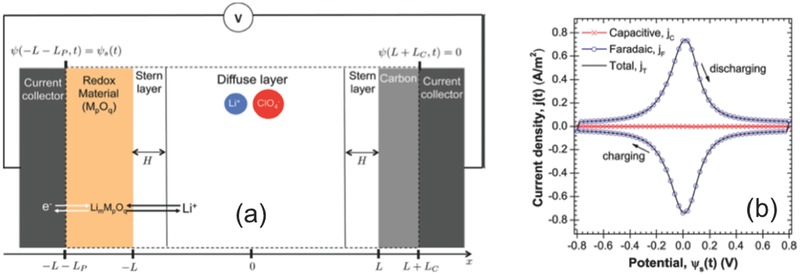
a) Scheme of a continuum model for simulating a lithium‐ion capacitor from numerically solving the transport model; b) simulated CV curve in the case of a thin‐film electrode with fast Li intercalation. Reproduced with permission.^242^ Copyright 2015, American Chemical Society.

Pilon et al. proposed a one‐dimensional continuum model to simulate the time‐dependent charging behavior of a hybrid capacitor consisting of a pseudocapacitive metal oxide electrode for lithium‐ion intercalation and a carbon electrode for EDL formation (**Figure** **25**a).^241^, ^242^, ^243^ Ion diffusion, lithium‐ion intercalation, and redox reaction were simultaneously captured. The numerically simulated CV graph showed good agreement with experiment. In the case of a thin‐film electrode of fast Li‐intercalation, the CV curve suggests that the Faradaic process dominates in the whole voltage range (Figure 25b). In the case of a thick oxide electrode where the kinetics is limited by Li intercalation, they found that the Faradaic region is at more negative potential, while the capacitive region is at high potential and dominated by the EDL formation and that the transition between Faradaic and capacitive behavior occurred between these two regions.^241^, ^242^, ^243^

### 7.2 Kohn‐Sham‐DFT‐Based Simulation for Pseudocapacitive RuO_2_ Electrodes

Besides intercalation pseudocapacitors, transition metal oxide can also store energy via surface redox reaction. To model this chemical behavior atomistically, electronic structure methods such as Kohn‐Sham DFT are necessary. The redox behavior of many kinds of metal oxide have been studied experimentally, such as RuO_2_, NiO, CoO_x_, MnO_x_, VO_x_ and FeO_x_.^244^, ^245^, ^246^, ^247^, ^248^ Among them, RuO_2_ is the most commonly studied system with the following redox chemistry in an acid electrolyte:^249^, ^250^, ^251^, ^252^, ^253^

$${\mathrm{RuO}}_{x}{\left(\mathrm{OH}\right)}_{y}+{\mathrm{zH}}^{+}+{\mathrm{ze}}^{-}\to {\mathrm{RuO}}_{x-z}{\left(\mathrm{OH}\right)}_{y+z.}$$

The oxidation state of Ru can be +2, +3, and +4, which can lead to a theoretical maximum capacitance over 1400 F g^–1^. Moreover, the charge storage mechanism of RuO_2_ depends on the scan rate: the CV curve shows a typical redox peak at low scan rate, but a rectangle shape at high scan rate, due to the transition from redox capacitive behavior to EDL capacitive behavior.^252^ Ozolins et al. investigated the charge‐storage mechanism of RuO_2_ with KS‐DFT^249^ on bulk proton insertion and surface proton adsorption. They found that RuOOH could stably exist and some subsurface of RuO_2_ were protonated in the voltage range of CV cycling, but the bulk diffusion of proton in RuO_2_ was found to have a high barrier. Their work suggested that the protonation reaction on surface and the particle boundary effect might be the key to interpret the pseudocapacitive behavior of RuO_2_.

**Figure 26:**
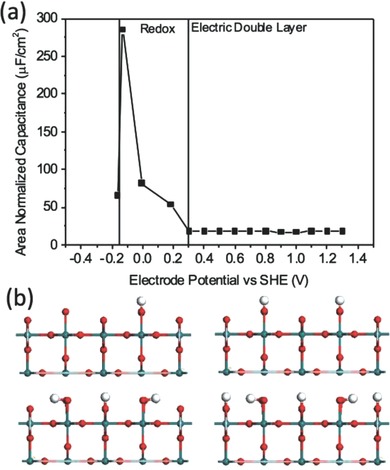
Pseudocapacitance of rutile RuO_2_(110) from joint DFT simulation with an implicit solvation model: a) differential capacitance; b) surface structures in the redox charging region (red, O; cyan, Ru; white, H). Adapted with permission.^254^ Copyright 2016, IOP Publishing.

To get a better understanding on the charge storage mechanism of RuO_2_, JDFT was applied to study the protonation reaction on the RuO_2_(110) surface in contact with an implicit solvation model.^254^ The simulation on neutral surface predicted a theoretical PZC that located just above the experimental redox peak, consistent with the reported experimental value. Simulation of the surface protonation reactions showed that hydrogen adsorption is energetically more preferable than EDL formation. A simple model was proposed to understand the pseudocapacitive behavior of RuO_2_: below the PZC, the capacitive behavior is governed by the hydrogen adsorption reaction on surface; above the PZC, the capacitive behavior is dominated by EDL formation. The pseudocapacitive region was computed by capturing the electronic chemical potential shift with hydrogen adsorption at different surface coverage. Simulated capacitance‐voltage curve (**Figure** **26**a) showed a qualitative agreement with experiment, indicating that the redox pseudocapacitance originates from the hydrogen adsorption on RuO_2_(110). In addition, the pseudocapacitance varies with hydrogen coverage and adsorption structure: at low coverage, the OH bond is perpendicular to the surface and the corresponding capacitance is relatively lower than that at high coverage with a bended OH bond (Figure 26b).^254^

## 8 Novel Material Design

Traditional materials for supercapacitors include mainly carbons, transition‐metal oxides, and redox‐reactive organic compounds. Novel materials such as MXene and borophene may have great potential in supercapacitor applications.

### 8.1 MXene Materials

**Figure 27:**
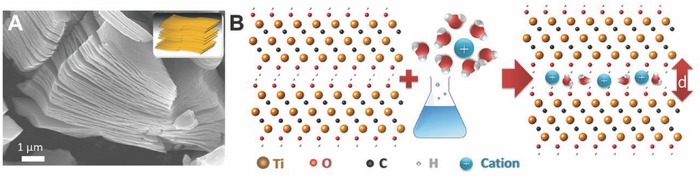
The structure of Ti_3_C_2_T_x_ layers and the scheme of cation intercalation. Reproduced with permission.^255^ Copyright 2013, the American Association for the Advancement of Science.

MXene materials have been experimentally synthesized and exhibit excellent capacitive performance. Gogotsi and coworkers reported the high volumetric capacitance of Ti_3_C_2_T_x_ (T = F, O, OH; **Figure** **27**) due to cation intercalation into MXene layers in aqueous electrolyte.^255^ The reported volumetric capacitance is over 300 F cm^–3^, much higher than that of porous carbon electrode. MXene was also suggested as an electrode material for lithium battery and intercalation capacitor.^256^ Xie et al. used KS‐DFT to study the lithium capacity of functionalized 2D MXene and found that the oxygen‐terminated group can provide the highest lithium capacity among other surface functional groups.^257^ Moreover, intercalation of MXene by other ions has been reported experimentally and theoretically.^258^, ^259^, ^260^ Oxygen‐terminated Ti_2_C was found to be a promising pseudocapacitive electrode due to its wide voltage window and low energy barrier for Na^+^ diffusion.^261^

Besides intercalation capacitance, MXene can also exhibit pseudocapacitance through surface redox reaction. Lukatskaya et al. studied the pseudocapacitive behavior of Ti_3_C_2_T_x_ (T = O and OH) in H_2_SO_4_ electrolyte.^262^ The pseudocapacitance of Ti_3_C_2_T_x_ was attributed to the redox behavior of Ti^3+/4+^ and surface protonation, similar to RuO_2_ pseudocapacitor (Figure 26). Wang et al. also reported on the pseudocapacitive mechanism of Ti_3_C_2_T_x_ in H_2_SO_4_ and revealed that both surface redox reaction and ion exchange contribute to capacitance.^263^

### 8.2 Boron Supercapacitors

**Figure 28:**
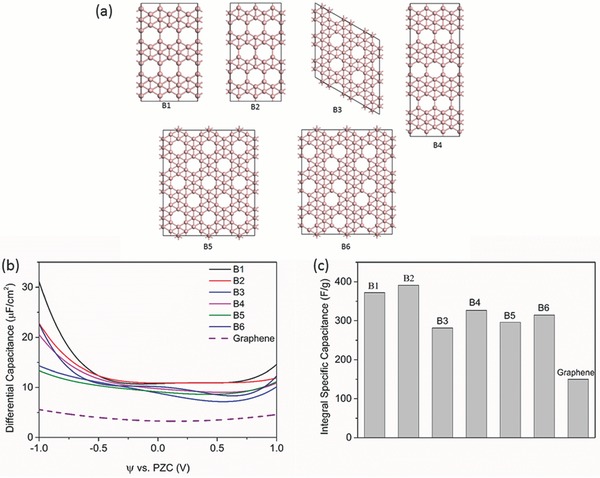
a) Structure, b) differential capacitance, and c) integral capacitance of 2D boron sheets from joint DFT simulation with an implicit solvation model. Reproduced with permission.^270^ Copyright 2016, American Chemical Society.

2D boron material was theoretically examined before,^264^, ^265^, ^266^, ^267^, ^268^ but the experimental realization of 2D boron sheet was just achieved recently.^269^ Moreover, many previous theoretical works showed that most 2D boron sheets are metallic. So they can serve as electrode materials for EDLCs. To demonstrate this idea, Zhan et al. theoretically investigated the capacitive performance of 2D boron sheets with JDFT, based on structures from early experimental and theoretical studies.^270^ They showed that 2D boron sheets have much higher areal and gravimetric capacitances than pristine graphene (**Figure** **28**). Although 2D boron sheet is not used in EDLCs yet, this prediction suggests that it has great potential in electrical energy storage.^270^

### 8.3 Other Novel 2D Materials for Energy Storage

Transition metal dichalcogenides (TMDs) are a popular class of 2D material that shows unique electronic property and catalytic activity in energy‐related application.^271^, ^272^ Especially, TMDs such as MoS_2_ have shown excellent performance in electrocatalysis, semiconductive physics, and energy storage. Their performance is dependent on the polytype (1T vs 2H phase) and can be modified by surface functionalization.^273^, ^274^, ^275^, ^276^, ^277^, ^278^ Chhowalla et al. showed that 1T phase MoS_2_ possesses very high volumetric capacitance from ≈400 F cm^–3^ to ≈700 F cm^–3^ in various electrolytes through ion intercalation from electrochemical measurement. They also showed that this material is suitable to high voltage window (3.5 V) and has stable performance over 5,000 cycles.^274^ However, the 2H phase of MoS_2_ only showed EDL capacitive behavior in an aqueous electrolyte.^279^, ^280^ Xie et al. reported that the metallic few‐layer VS_2_ exhibits the capacitance of 4760 µF cm^–2^ (317 F cm^–3^) with the cycling time over 1000.^281^ Dunn et al. measured the capacitance of TiS_2_‐T at ≈320 F g^–1^ in a lithium‐ion electrolyte.^282^ The intrinsic charge storage capability of TMDs was systematically predicted with KS‐DFT calculations.^283^

## 9 Summary and Outlook

In this review, we summarized recent computational insights into capacitive energy storage to address several important issues in supercapacitors especially EDLCs. The fundamental physics of EDLCs is ion separation and sorption on the electrode surfaces. Built upon conventional interfacial double‐layer theory (Helmholtz, GC and GCS models), a more accurate interfacial description has been obtained analytically,^10^, ^12^, ^13^ in terms of the shape of the differential capacitance vs potential for different ionic concentrations next to a planar electrode. Coarse‐grained models, such as CDFT and coarse‐grained MD or GCMC, provided microscopic insights into the ionic response and EDL structure more accurately than analytical modeling.^65^, ^143^ In particular, CDFT allows one to efficiently examine how the ion size, ion valence, ion concentration, and the electrode pore size/geometry influence the capacitive performance. The atomic level information of the electrolyte response behavior could be obtained from CMD with all‐atom force fields. With this tool, one could study the influence of electrode geometry and electrolyte chemistry on the capacitive performance. Moreover, CMD is also applicable to more realistic and complex porous carbon models with the constant potential method.^101^ Thus, the capacitance determined by electrolyte property and electrode geometry can be well understood by the classical simulation techniques (CDFT, GCMC and CMD). Charging kinetics can be simulated by time‐dependent CDFT, while CMD can provide insights into how pore size impacts ion diffusivity at different electrode potentials.

To capture the role of electrode chemistry, the electronic‐structure methods such as Kohn‐Sham DFT are necessary and the key is to integrate the electronic structure contribution with the EDL contribution to obtain the total or device capacitance. In the case of 2D materials of low electronic DOS at the Fermi level such as graphene, the electrode contribution is reflected in the quantum capacitance (C_Q_). C_tot_ of the electrode/electrolyte interface can be modeled separately by electronic‐structure‐derived C_Q_ and classically obtained C_EDL_.^80^ This method of combining C_Q_ and C_EDL_ showed good agreement with experimentally measured capacitance for the single‐layer graphene electrode. Beyond this two‐contribution model (C_Q_ and C_EDL_), the third contribution C_Dielec_ was revealed by both ESM and JDFT methods, which could capture the influence of dielectric screening of the electrode region in total capacitive behavior in few‐layer‐graphene electrodes.^129^, ^222^ Consequently, the charge‐capacitive mechanism of EDLCs can be treated as a joint consequence of electrode (C_Q_ and C_Dielec_) and electrolyte (C_EDL_) behavior during charging/discharging.

The strategy to improve the performance of porous carbon EDLCs can be considered from two aspects: (i) increasing C_Q_ and decreasing dielectric screening contribution through electrode modification (thickness and size control, heteroatom doping, topological defects, edge plane fabrication, and surface functionalization); (ii) electrode geometry and electrolyte property modification to enhance the counterion sorption and coion exclusion (slit pore size, pore geometry, pore size distribution, ion size, valence, concentration and solvent property). Many simulations have predicted higher capacitances along those lines.

Different from EDLCs, surface redox and ion‐intercalation pseudocapacitors are more complicated and difficult to simulate. A 1D continuum transport model was used to describe the hybrid pseudocapacitor based on the lithium intercalation and diffusion in metal oxide,^241^ while JDFT was applied to study the protonation reaction and surface redox pseudocapacitance of RuO_2_ (110) surface.^254^ More recently, Keilbart et al. proposed a method that combines Kohn‐Sham DFT with continuum solvation models and Monte Carlo to simulate the pseudocapacitive response of RuO_2_ surface in the H_2_SO_4_ electrolyte.^284^

The capacitive behavior stems from the electrolyte response to the electrode surface charging: charged electrode causes the electrolyte response through ionic screening and polar solvent response; then the rearranged electrolyte structure polarizes the electrode and affects the electronic distribution. Constant charge CMD with all‐atom force field can accurately predict the electrolyte response, but the electrode charge distribution is not optimized. Constant potential MD and MC were proposed in recent years, but could not capture the electrode polarization. Electronic DFT coupled with a solvation model, such as JDFT and ESM, can capture the polarization of electrode from ionic screening in the electrolyte at the electronic‐structure level, but the electrolyte response is limited by the implicit solvation model. Moreover, the computational cost can be prohibitive for the solvation‐embedded DFT code due to the expensive computation for the solid/liquid coupling, especially in ESM.

Another difficulty in EDLC modeling is the influence of surface chemistry. When the surface functional group could react with the ions or solvent, only considering the electrostatic interaction is not enough to capture the capacitive behavior. So how to take into account the surface chemistry in EDLC modeling is also a challenging and unsolved issue, since it blurs the line between EDLC and pseudocapacitor. Modeling pseudocapacitor is still in its infancy. KS‐DFT study can provide insights into the micro‐dynamics of lithium diffusion and transport in the oxide. For the pseudocapacitor governed by the surface redox reaction, currently there is no good model to describe it due to the complexity of the interfacial physical chemistry. A typical case is the transition metal oxides in an acid electrolyte: the surface structure, surface reaction pathway, reaction kinetics, over‐potential, solvent effect and electrolyte pH are all able to greatly influence the pseudocapacitive behavior.

In recent years, combining two or more types of conventional electrode materials to make hybrid electrodes also became a promising way to improve the capacitive performance of supercapacitor. One typical case is the pseudocapacitive polypyrrole/MXene hybrid electrode.^262^ Another typical example of a hybrid electrode is based on 2,5‐dimethoxy‐1,4‐benzoquinone on reduced graphene‐oxide sheets.^285^ For these hybrid systems, theoretical simulation can be extremely difficult due to the complex capacitive mechanism coupling together surface redox, ion intercalation, ion diffusion and EDL formation at the same time. Thus, a step‐by‐step and multi‐scale approach is necessary.

## Conflict of Interest

The authors declare no conflict of interest.
